# Simulation-based inference of differentiation trajectories from RNA velocity fields

**DOI:** 10.1016/j.crmeth.2022.100359

**Published:** 2022-12-19

**Authors:** Revant Gupta, Dario Cerletti, Gilles Gut, Annette Oxenius, Manfred Claassen

**Affiliations:** 1 Internal Medicine I, University Hospital Tübingen, Faculty of Medicine, University of Tübingen, Tübingen, Germany; 2 Department of Computer Science, University of Tübingen, Tübingen, Germany; 3 Institute of Molecular Systems Biology, ETH Zurich, Zurich, Switzerland; 4 Institute of Microbiology, ETH Zurich, Zurich, Switzerland

**Keywords:** single-cell RNA sequencing, RNA velocity, trajectory inference, simulation-based inference

## Abstract

We report Cytopath, a method for trajectory inference that takes advantage of transcriptional activity information from the RNA velocity of single cells to perform trajectory inference. Cytopath performs this task by defining a Markov chain model, simulating an ensemble of possible differentiation trajectories, and constructing a consensus trajectory. We show that Cytopath can recapitulate the topological and molecular characteristics of the differentiation process under study. In our analysis, we include differentiation trajectories with varying bifurcated, circular, convergent, and mixed topologies studied in single-snapshot as well as time-series single-cell RNA sequencing experiments. We demonstrate the capability to reconstruct differentiation trajectories, assess the association of RNA velocity-based pseudotime with actually elapsed process time, and identify drawbacks in current state-of-the art trajectory inference approaches.

## Highlights

• Trajectory inference leveraging RNA velocity with no topological constraints
• Cytopath models complex behavior like cycles, convergences, and interlaced processes
• Pseudotime ordering inferred by Cytopath approximates rate of change

## Motivation

Trajectory inference from single-cell RNA sequencing data has the potential to systematically reconstruct complex differentiation processes, but inferring trajectories that accurately model the biological characteristics of varied processes continues to be a challenge, regardless of the many available solutions. In general, trajectory and pseudotime inference methods have so far suffered from the ambiguity of static single-cell transcriptome snapshots lacking a concept of directionality and rate of transcriptional activity.

## Graphical abstract

**Figure:**
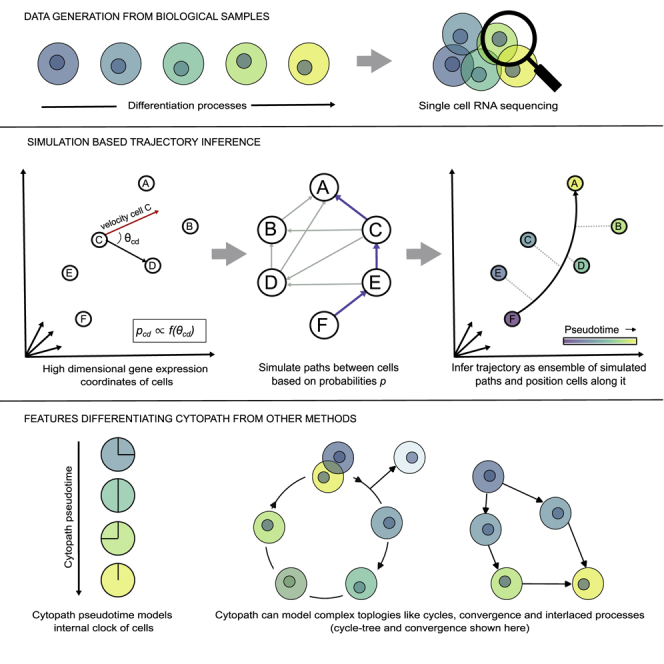

## Teaser

Gupta et al. develop a method to model differentiation processes with single-cell RNA sequencing data that has the capacity to model complex behaviors like cycling and convergence as well as co-occurring combinations of multiple processes.

## Introduction

Biological processes such as cell type differentiation,^1^^,^^2^^,^^3^ immune response,^4^ or cell division^5^ can be conceptualized as temporal sequences of coordinated, phenotypic state changes in the context of possibly heterogeneous cell populations. Such phenotypic states can be characterized by, e.g., epigenetic, transcriptional, and proteomic cell profiles. These differentiation processes are often triggered asynchronously. The differentiation processes give rise to state sequences with varying topologies, including bifurcating, multi-furcating, cyclical, and convergent trajectories.

This situation requires single-cell approaches to measure and ultimately investigate these processes. The repertoire of suitable technologies to monitor different types of molecular profiles has increased dramatically over the last years. In particular, single-cell RNA sequencing (scRNA-seq) has gained widespread use because of the broad applicability of sequencing technology. Although these measurements are information rich, their analysis and interpretation are challenged by high dimensionality, low sequencing depth, measurement noise, and its destructive nature, only yielding snapshots of the whole process.

Different computational approaches have been proposed to model differentiation processes from scRNA-seq data, specifically covering the tasks of pseudotime estimation, trajectory inference, or cell fate prediction. These tasks are related but typically require different approaches (Table S1). The goal of cell fate prediction is to determine the terminal differentiation state (fate) of any cell, possibly already early in the differentiation process. Such methods generate a score or probability per cell with respect to terminal differentiation states.^6^^,^^7^

Pseudotime estimation addresses the task of ordering observed cells into a sequence of cell states traversed by a differentiation process. Typically, the estimated pseudotime values are interpreted as temporal ordering, not capturing the pace of differentiation. It has been suggested that RNA velocity-based pseudotime has the potential to overcome this limitation.^8^ Although pseudotime estimation might constitute sufficient characterization of a linear differentiation process, the description of complex processes with more involved topologies, such as bifurcations, requires an additional step of trajectory inference. Trajectory inference methods seek to infer a representative sequence of states that characterizes the possibly multiple differentiation processes in branching or convergent differentiation.^9^^,^^10^^,^^11^^,^^12^

Typical trajectory inference methods are guided by the assumption that phenotypic similarity reflects temporal proximity. However, static expression profiles are ambiguous with respect to the directionality of potential cell state transitions. This ambiguity is a major limitation of pseudotime ordering and trajectory inference and specifically precludes data-driven assignment of root and terminal states without previous knowledge about the process as well as resolving complex (i.e., cyclical^5^ or convergent^3^) process topologies. It has now become possible to estimate transcriptional activity from scRNA-seq data via RNA velocity analysis,^1^ enabling inference of likely transitions between different cell states in a data-driven fashion, ultimately opening the possibility to mitigate the limitations of the aforementioned reconstruction approaches.

In this work we present Cytopath, a simulation-based trajectory inference approach that takes advantage of RNA velocity. We demonstrate that Cytopath infers accurate and robust cell state trajectories of known differentiation processes with linear, circular, bifurcated, tree-like, and convergent topologies from scRNA-seq datasets. We show that Cytopath has the potential to model interlaced processes with different topologies as well as detect regions of transcriptional program switching. We also assess the ability of pseudotime estimated by Cytopath to represent the biological process time, also referred to as the “internal clock” of a cell. Trajectory inference with Cytopath addresses the limitations of state-of-the-art trajectory inference approaches as well as recently developed RNA velocity-based methods.^11^^,^^12^

## Results

Here, we present an overview of Cytopath and its trajectory inference performance, assessed on six scRNA-seq datasets consisting of cellular differentiation processes with various topologies.^1^^,^^2^^,^^3^^,^^4^^,^^5^^,^^13^ We compare the performance of Cytopath with the best trajectory inference models for each topology:^14^ Slingshot^9^ for tree-like topology, Angle^14^ for cell cycle, and partition-based graph abstraction (PAGA; directionality enabled by velocity pseudotime)^8^^,^^15^ for graph models. We also include a comparison with Monocle3^16^ as well as two approaches accounting for RNA velocity information: VeTra^11^ and Cellpath.^12^

### Simulation-based trajectory inference with Cytopath

**Figure 1:**
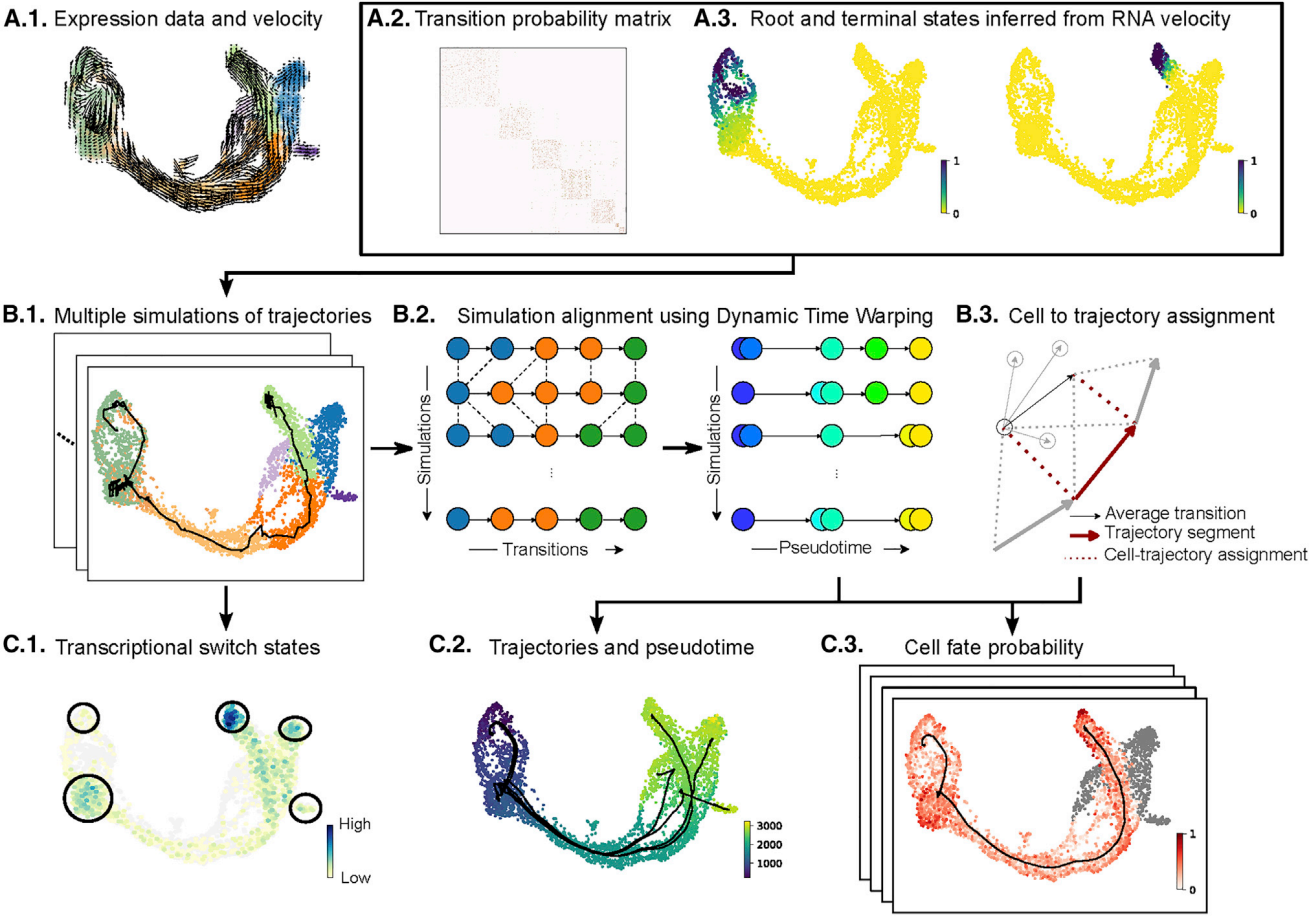
Cytopath overview (A) Inputs for Cytopath trajectory inference subsequent to an RNA velocity analysis (shown here: inferred using RNA velocity). (A.1.) Single-cell gene expression profiles and RNA velocity profiles. (A.2.) Transition probability matrix. (A.3.) Root and terminal state annotation. (B) Steps performed during Cytopath inference. (B.1.) Simulations of the differentiation process generated by sampling a Markov chain based on the cell-to-cell transition probabilities. Sampling is initialized on cells annotated as root states. (B.2.) Simulations are performed for a fixed number of steps that are automatically selected using the properties of the scRNA-seq dataset. Transition steps are aligned using dynamic time warping. After alignment, cells at each transition step represent the same consensus state. (B.3.) Cells along the inferred trajectory are assigned to multiple trajectory segments based on the alignment of their average transition vector (with respect to neighbors) and the trajectory segment. (C) Outputs from Cytopath trajectory inference. (C.1.) The frequency of simulations terminating at each cell highlights regions of switch in transcriptional programs as well as terminal regions. (C.2.) Trajectories are inferred independently for each terminal region. The trajectories are composed of multiple segments. The pseudotime of a cell is estimated as the weighted average segment rank of all segments with which it aligns. (C.3.) Differential alignment scores to multiple trajectories are used to estimate the cell fate probability with respect to the terminal regions.

Trajectory inference with Cytopath is performed downstream of the RNA velocity analysis of an scRNA-seq dataset and is specifically based on the resulting cell-to-cell transition probability matrix. The transition probability matrix considers each cell to be a node in a graph, and each node is assumed to represent a possible state of the differentiation process under study. The entries of this matrix are the probabilities of transitioning from a given state to any other state represented in the graph.^1^^,^^8^ Although we base our analysis on an RNA velocity analysis, in principle, any cell-to-cell transition probability matrix can be used as input for trajectory inference (Figure 1A.2).

The objective of trajectory inference with Cytopath is to estimate trajectories from root to terminal cell states, which correspond to the origin and terminus of the differentiation process under study. Root and terminal states can be derived from a Markov random-walk model utilizing the transition probability matrix itself (Figure 1A.3), as described by La Manno et al.,^1^ or can be supplied by the user based on suitable prior knowledge.

The trajectory inference process is divided into four steps (Figure 1B; STAR Methods). In the first step, Markov sampling of consecutive cell state transitions is performed based on the probabilities of the transition matrix, resulting in an ensemble of simulated cell state sequences. Sampling is initialized at predefined root states and performed for a fixed number of steps until a sufficient number (auto-selected with default settings) of unique cell state sequences terminating within clusters containing the terminal states have been generated (Figure 1B.1). A pre-computed clustering can be provided to Cytopath to determine terminal regions; otherwise, a clustering is computed internally using Louvain.

The generated cell state sequences are individual simulations of the differentiation process from root to terminal state. Because of the stochastic nature of the sampling process, the cell state sequences cannot be considered aligned with respect to the cell states at each transition step. Consequently, in the second step, simulations that terminate at a common terminal state are aligned using dynamic time warping, an algorithm for comparing and aligning temporal sequences with a common root and terminus but possibly different rates of progression. The procedure aligns simulations with a common differentiation coordinate so that cell states from any simulation at a particular differentiation coordinate (pseudotime) represent similar cell states (Figure 1B.2).

Third, consensus expression states across the steps of the aligned simulations are estimated, giving rise to the reported trajectory. Cell states at every step of the ensemble of aligned simulations are averaged, and the average value is considered the consensus state of the trajectory at the particular step (Figure 1C.2). Alternatively, trajectories can be anchored to observed cell states by choosing the cell state closest to the aforementioned average value. Subsequently, the coordinates of the trajectory with respect to the expression space as well as any lower dimensional embeddings, such as UMAP or t-SNE, are calculated.

In the final step, cells are assigned to each step of the inferred trajectory. Assignment is based on an alignment score that evaluates for each cell the similarity of its static as well as the velocity profile with each trajectory step. For efficiency, this alignment score evaluation is restricted to cells in the neighborhood around each trajectory step. However, the user can optionally compute alignment of every cell for every step of every trajectory. The cell level score is used to estimate position in the trajectory (i.e., pseudotime) as well as the relative association of a cell state to possibly multiple branches of a differentiation processes with complex topology (i.e., cell fate) (Figures 1C.2 and C.3).

### Reconstruction of neuronal differentiation in the developing mouse hippocampus

We assessed the capability to reconstruct developmental processes with multiple branching, which is a frequent topology for scRNA-seq datasets generated from experiments studying differentiation processes. To this end, we applied Cytopath and baseline methods to the developing mouse hippocampus dataset, which was first used to demonstrate RNA velocity of single cells. This dataset is composed of 18,140 cells. The dataset comprises five terminal regions and a common root state. The topology of the data is multi-furcating, with development branches arising directly from the root state (astrocytes and oligodendrocyte precursors [OPCs]) but also as branches from intermediate states (neuroblast and Cajal-Retzius [CA] differentiation).

We recreated the analysis outputs, including RNA velocity and the transition probability matrix, as indicated in the original publication, using scripts made available by the authors. RNA velocity was used to estimate the root and terminal state probabilities.^1^

**Figure 2:**
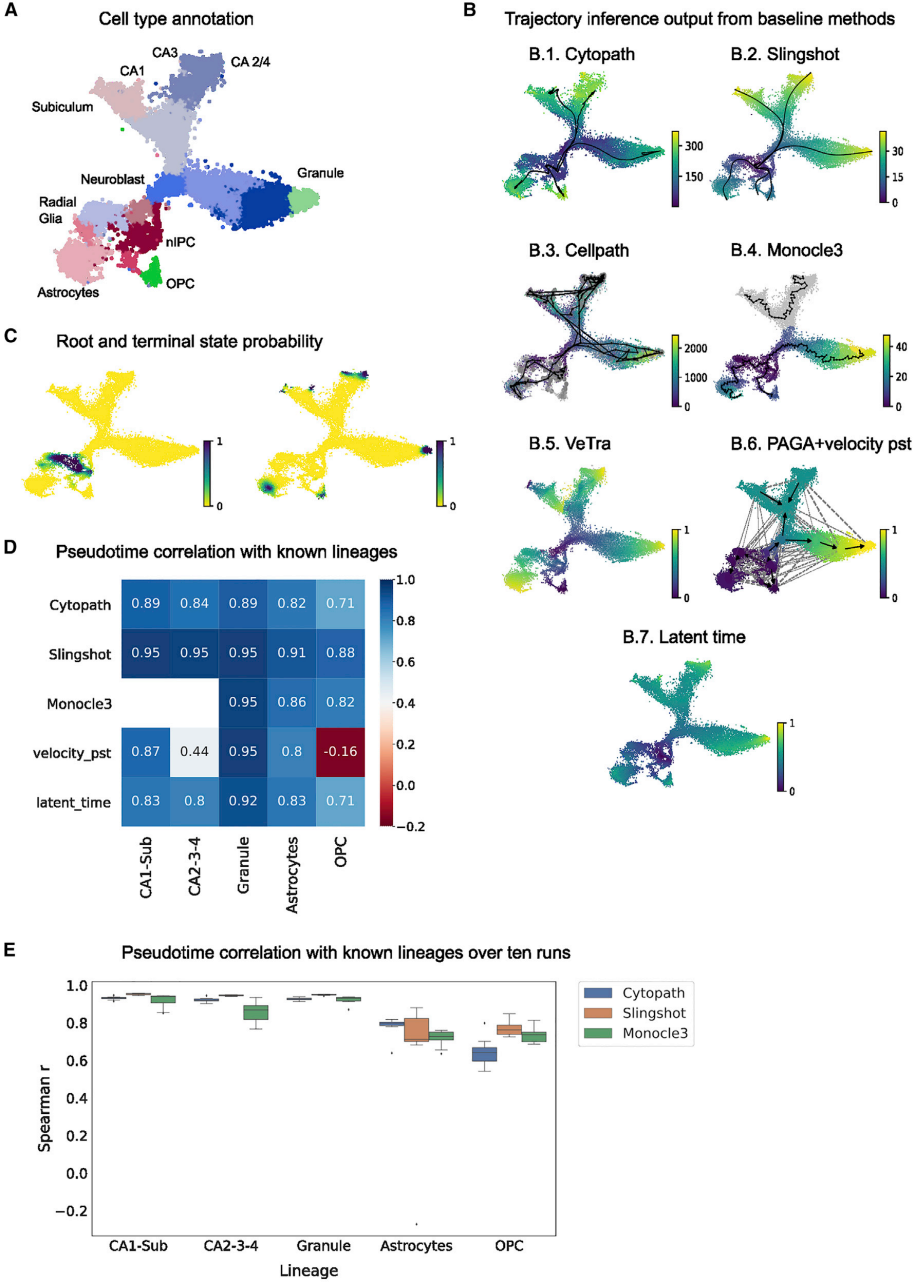
Reconstruction of neuronal differentiation in the developing mouse hippocampus (A) t-SNE projection of the dentate gyrus scRNA-seq dataset annotated with stages of neuronal differentiation. (B) Trajectory and/or pseudotime inference using (B.1.) Cytopath, (B.2.) Slingshot, (B.3.) Cellpath, (B.4.) Monocle3, (B.5.) VeTra, (B.6.) PAGA + velocity pseudotime (vpt), and (B.7.) scvelo latent time. (C) Root and terminal state probability used by Cytopath to select root and terminal regions. (D) Spearman correlation of pseudotime inferred by each method with known ordering of cell types for each lineage. (E) Methods were run 10 times to assess the effect of stochasticity in inference (Cytopath) and stochastic estimation of the UMAP embedding (Slingshot and Monocle3). Monocle3 produced disconnected graphs in two of 10 runs corresponding to the CA1-Sub and CA2-3-4 lineages, and the correlation value could not be calculated for these two runs.

Spearman correlation of inferred pseudotime with the cell type identities and their ordering reported in the initial study (Figure 2A) were used for trajectory inference performance assessment (Figures 2D and 2E). Cytopath was run using default parameters. It internally selects root and terminal states based on the root and terminal state probabilities estimated in the prior velocity analysis. We also supply the known root and terminal states as supervision to Slingshot and Monocle3 (which accepts root states only) to get the best performance from these methods. This could not be done for VeTra and Cellpath because these approaches do not allow inclusion of this supervision. We also assessed the performance of PAGA with velocity-pseudotime-based directionality and scvelo latent time. The latter two are not trajectory inference methods; PAGA only generates a coarse graph of cluster connectivities, and latent time is only a pseudotime that does not compute lineage association of cells. However, as RNA velocity-based methods that have a partial overlap with Cytopath’s core functionality, this comparison is of interest to the community.

Trajectories and pseudotime estimated by each method are shown in Figure 2B. Cytopath estimates a single trajectory to each terminal state as expected from known biology. The Spearman correlation between pseudotime inferred by Cytopath for each trajectory to known ordering of cell types is high (Figure 2D) and robust across multiple runs (Figure 2E). Slingshot also produces trajectories to each terminal state with high correlation but generated one or more spurious trajectories in each run. Although the median Spearman correlation of trajectories inferred by Slingshot is high, it appears to have high variability in its performance, with substantially lower correlation for some runs. This may be due to projection artifacts in the embedding generated in those instances. Monocle3 fails to produce a connected trajectory, producing a disjoint graph, and is therefore unable to estimate pseudotime for a large portion of the dataset. Monocle3 pseudotime, velocity pseudotime, and latent time are inferred per cell, and, unlike other methods presented here, do not partition cells into trajectories. We used known cell type ordering to select cells relevant to each lineage to perform the correlation analysis.

The velocity-pseudotime method appears to compute a global pseudotime that is incompatible with known differentiation of lineages in this dataset. Consequently, the directionality of cluster transitions for PAGA appears to be reversed for CA1-Sub and CA2-3-4 lineages and is unclear for OPC and astrocyte lineages. The latent time method appears to have a similar correlation profile to known lineages as Cytopath (Figure 2D).

VeTra and Cellpath infer erroneous trajectories that initiate at terminal or intermediate states. Cellpath generates a large number of trajectories far exceeding the number of known lineages. This is a pattern that is consistent across several datasets; thus, a quantitative comparison as performed for other methods presented here is not feasible (Figure S1). Both methods also exclude a large number of cells from the trajectory inference process. Because VeTra and Cellpath initialize trajectories in intermediate states, cell assignment by these methods does not correspond to a pattern expected for a hierarchical branching structure (Figures S2A.3 and A.4).

### Cell cycle reconstruction

We hypothesized that the ability to infer repeating patterns during differentiation likely differentiates RNA velocity-based trajectory inference from other methods that are based on similarity of expression. First, revisiting the root state implies that inferring the overall direction of the trajectory is not trivial. Second, cells at the origin are a mix of late- and early-stage states that are co-located in expression space but can be expected to have differing velocities. To assess this hypothesis, we compared the reconstruction of the cell cycle in a dataset comprising 1,067 U2OS cells generated using the SMART-Seq2 protocol.^5^

**Figure 3:**
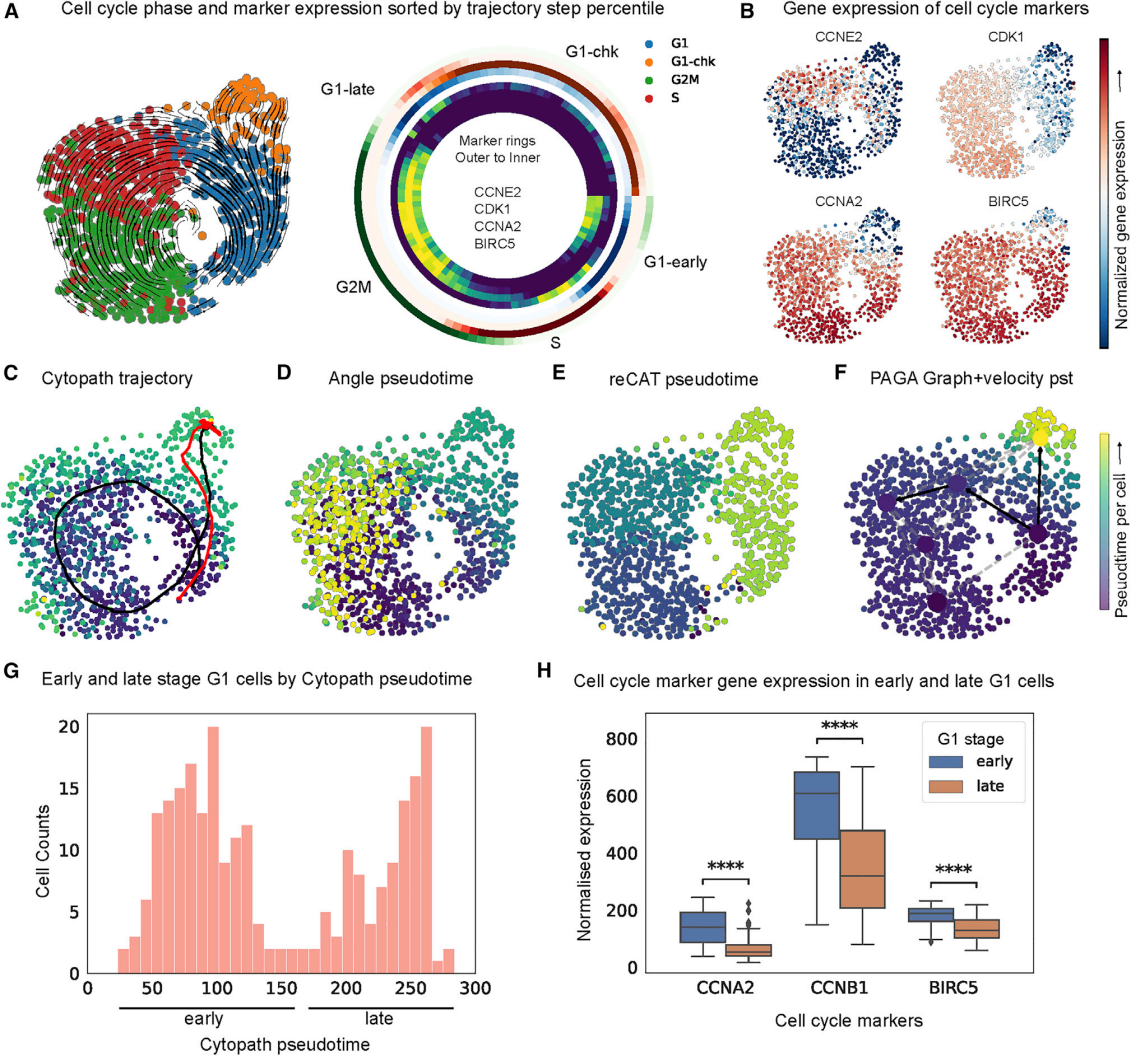
Reconstruction of the cell cycle in the U2OS cell line (A) RNA velocity stream plot overlayed on the UMAP projection, annotated with the cell cycle phase adapted from Mahdessian et al.^5^ Considering all cell-to-trajectory alignments binned into percentiles, the radial heatmap shows cell cycle phase fraction (outer set of rings) and marker expression (inner set of rings) sorted by trajectory step. The directionality of the radial heatmap is clockwise, with the origin at zero degrees. (B) The separation of G1 phase into G1 and G1-chk was performed on the basis of marker expression of cell cycle genes. (C–F) Trajectories inferred and pseudotime per cell by (C) Cytopath, (D) Angle, (E) ReCAT, (F) PAGA and vpt. (G) Distribution of Cytopath pseudotime for cells in the G1 cluster. (H) Normalized expression of cells classified as early and late G1 cells (blue/orange, respectively). Significance was estimated by an independent t test for each marker.

Based on the comparatively low expression of the cell cycle marker genes *Ccne2*, *Cdk1*, *Ccna2*, and *Birc5* (Figures 3A and 3B), we annotated a portion of G0-stage cells (cluster 5) as a G1 checkpoint (Figure S4A). The cell cycle phase annotation per cell from Mahdessian et al.^5^ was determined using the fluorescence intensity of GFP-tagged GMNN (530 nm) and RFP-tagged CDT1 (585 nm). Therefore, the association between cell cycle phase and expression levels of markers is not in phase, unlike computational cell cycle phase prediction (Figures S4B and S4C). We use phase annotations only to validate the trajectory reconstruction but not for inference. Root and terminal states were selected based on probabilities estimated using scvelo.^8^

Cytopath generates a full circular trajectory without interruptions from the cells in G1 stage through the intermediate stages back to the G1 stage and further into the cells in the G1 checkpoint stage (Figure 3C). The lower expression of relevant cell cycle markers in the terminal region of the trajectory inferred by Cytopath indicates that the pseudotime inferred by Cytopath is a valid representation of the temporal process. Cytopath infers a second linear trajectory, indicated in red (Figure 3C). This is not unexpected because, apart from the circular route, a direct connection of the root to the terminal region is also a possible outcome.

Because Slingshot and Monocle3 assume a tree-like topology, they are inherently unsuited to model cyclical trajectories. To compare Cytopath with non-velocity-based baseline methods, we selected Angle and ReCAT, methods intended to model the cell cycle in scRNA-seq data. In contrast to trajectories inferred by Cytopath, the pseudotime inferred by Angle and ReCAT is inconsistent with marker expression and cell cycle phase annotation. Both methods fail to correctly model the cell cycle, possibly because of the presence of G1 checkpoint cells that are not actively participating in the cycling process. Although pseudotime inferred by Angle represents a partially correct sequence of clusters, the G1 checkpoint cluster is not distinguished from the G1 cluster. S phase is incorrectly identified as the terminal state. ReCAT successfully identifies the G1 checkpoint as the terminal state but detects an incorrect sequence of cell cycle phases (Figures 3D and 3E).

With respect to the second part of our hypothesis, we observe that cells in the G1 cluster can be divided into two groups on the basis of pseudotime inferred by Cytopath (Figure 3G). Expression of markers associated with cell cycle is significantly higher in early-pseudotime G1 cells than in those destined to move into G1 checkpoint phase and, accordingly, associated with higher pseudotime (Figure 3H). After trajectory inference, cells are assigned to trajectories using the alignment procedure shown in Figure 1B.3. We sort these cell-to-trajectory alignments by trajectory step percentile and then compute cell cycle phase frequency and average marker expression. Partitioning of G1 cells into early and late stage as well as the difference in marker expression can be clearly observed as two separate bands of G1 (blue) in the radial plot (Figure 3A).

We assessed PAGA with directionality inferred using velocity pseudotime (Figure 3F). PAGA failed to estimate an unbroken sequence of cluster transitions with a default threshold (connections in black), and when the entire connectivity graph is considered (all connections), there are several spurious connections. Although the underlying velocity pseudotime is positively correlated with cell cycle phase, G1 cells are not partitioned into early- and late-stage states. Latent time also correctly models the sequence of cell cycle phases and identifies G1 checkpoint phase as the terminus. However, similar to velocity pseudotime, G1-phase cells are not partitioned into early- and late-stage states (Figure S3B.3).

Finally, VeTra and Cellpath, which are RNA velocity-enabled trajectory inference methods, fail to correctly reconstruct the cell cycle. The pseudotime inferred by VeTra appears to be inconsistent with the root and terminal state probabilities. Both methods infer erroneous trajectories that do not capture the cyclical process, both originating and terminating in intermediate states. Both methods do not assign large number of cells to any trajectory (Figures S1B.3, S1B.4, S2B.3, and S1B.4).

### Reconstruction of interlaced cell cycling and bifurcated differentiation in pancreatic endocrinogenesis

We next assessed trajectory inference performance for processes with multiple interlaced non-trivial topologies. To this end, we considered a dataset studying pancreatic endocrinogenesis with lineages to four terminal states (alpha, beta, gamma, and delta cells) and dominant cell cycling at the onset of differentiation.^2^^,^^8^ Pre-processing, RNA velocity, and transition probability matrix estimation were performed with scvelo^8^ using parameters indicated in the notebook associated with this dataset.

**Figure 4:**
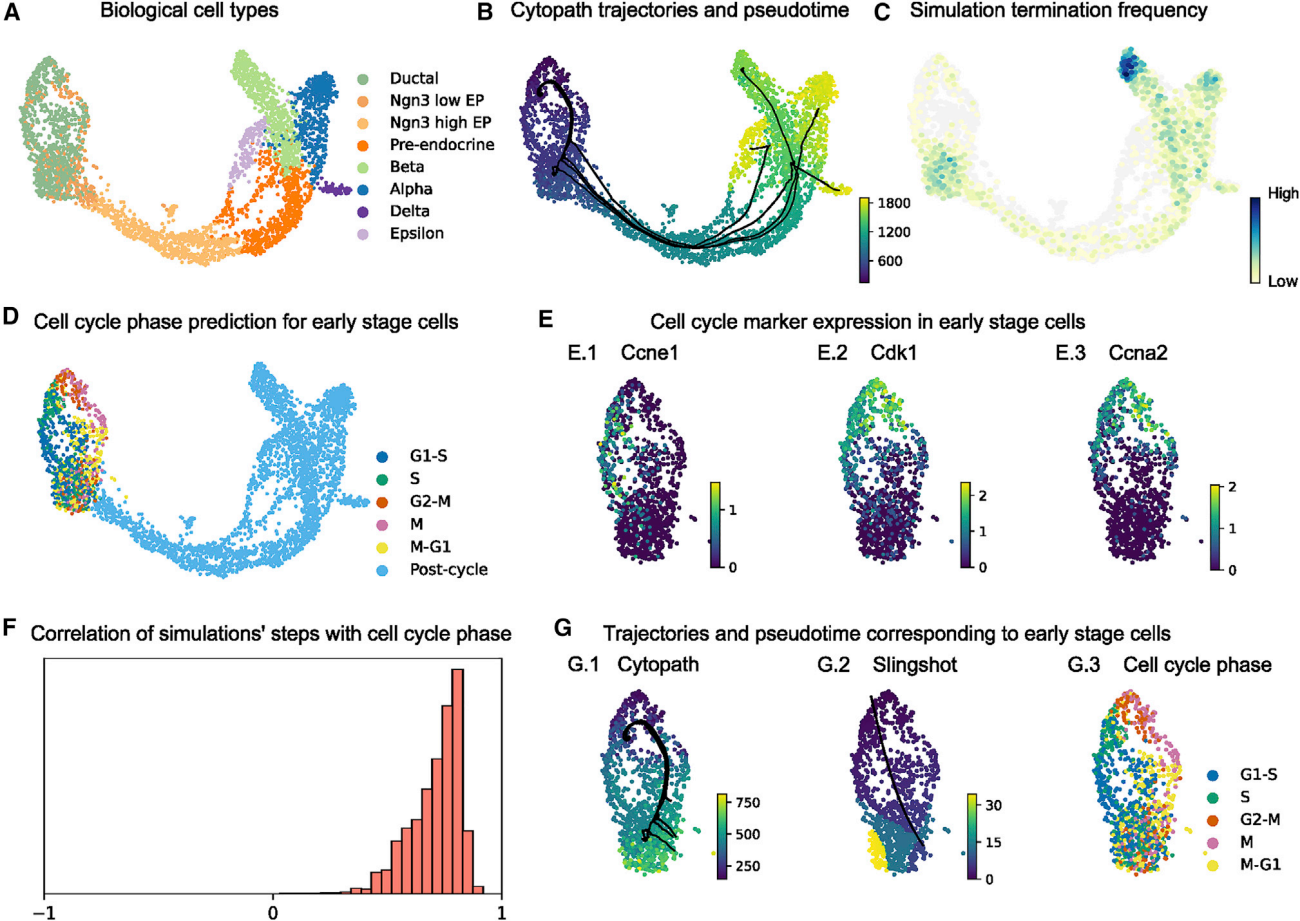
Reconstruction of interlaced cell cycling and bifurcated differentiation in pancreatic endocrinogenesis (A) UMAP projection of pancreas scRNA-seq data annotated with stages of differentiation. (B) Trajectories inferred by Cytopath and mean pseudotime per cell. (C) Log terminal state frequency per cell of undirected simulations initialized at randomly chosen cells. (D) Computational cell cycle phase prediction. (E) Cell cycle marker gene expression in the early stage (Louvain clusters 9, 0, and 4) (Figure S4D). (F) Spearman correlation of cell cycle phase with the transition step of individual Cytopath simulations. (G) Trajectories and pseudotime inferred by (G.1) Cytopath and (G.2) Slingshot in the root region and (G.3) cell cycle phase annotation.

Cell type annotation from Bastidas-Ponce et al.^2^ and Bergen et al.^2^^,^^8^ was used to provide terminal state supervision to Cytopath (Figure 4A), whereas root states were inferred using RNA velocity. The inferred terminal state probability only identifies the beta terminal state (Figure S5A3). If the other terminal states are not manually specified, then this exclusively data-driven approach would report only the trajectory corresponding to the beta lineage. However, Cytopath can be used to generate undirected simulations not constrained to terminate at a fixed terminal region (STAR Methods). For each cell, the frequency of simulations terminating at that state can be used to discover regions of transcriptional state switching. Using this approach, two more terminal states (alpha and delta) could be recovered (Figure 4C). However, the trajectory to the epsilon terminal state could only be constructed by explicitly providing it as a terminal state. The set of trajectories estimated by Cytopath corresponding to the four terminal cell types captures the expected differentiation events of endocrinogenesis (Figure 4B).

The trajectories visualized on the UMAP indicate a potentially cycling structure in early-stage (root-region) cells. To investigate this, we initialized simulations at random cells in the dataset (Figure 4C). We observed an enrichment of terminal states of these undirected simulations in Louvain cluster 0 (Figure S4D). We propose that this observation suggests that this differentiation process is structured in two stages, a cycling and a commitment stage, with the cells in Louvain cluster 0 corresponding to a region of transcriptional switch away from cell cycling. The following inquiries aim to identify evidence for this hypothesis.

Cell cycle scoring of cells in the root region was performed and clearly revealed distinct cell cycle states (Figure 4D). This interpretation is also supported by the differential expression of cell cycle marker genes in the root region (Figure 4E). The trajectory inferred by Cytopath from the ensemble of 8,000 simulations appears to recapitulate the circular structure of the cell cycle (Figure 4G1). Spearman correlation of cell cycle phase with the transition steps of each simulation indicates faithful recapitulation of the cell cycle stages at the single-simulation level (Figure 4F).

The simulation-based approach of Cytopath ensures that, even in the absence of explicit supervision, cyclic transcriptional patterns are reconstructed faithfully. In contrast, possibly because of the absence of RNA velocity information, the designated root states appear to be isotropic for conventional trajectory inference approaches like Slingshot; therefore, they are unable to capture structured transcriptional heterogeneity in this region (Figure 4G.2).

We also show the trajectory estimation with respect to the full pancreatic endocrinogenesis process. Slingshot and Monocle3 produce spurious or too few trajectories, respectively, when provided with all root and endpoints. VeTra reports a spurious trajectory that terminates at the ductal stage, whereas trajectories to beta and alpha are initialized in intermediate or terminal cell states. VeTra and Cellpath exclude a large number of cells from the trajectory inference process (Figures S1C, S2C, and S3C).

### Reconstruction of convergent differentiation in the developing neonatal mouse inner ear

**Figure 5:**
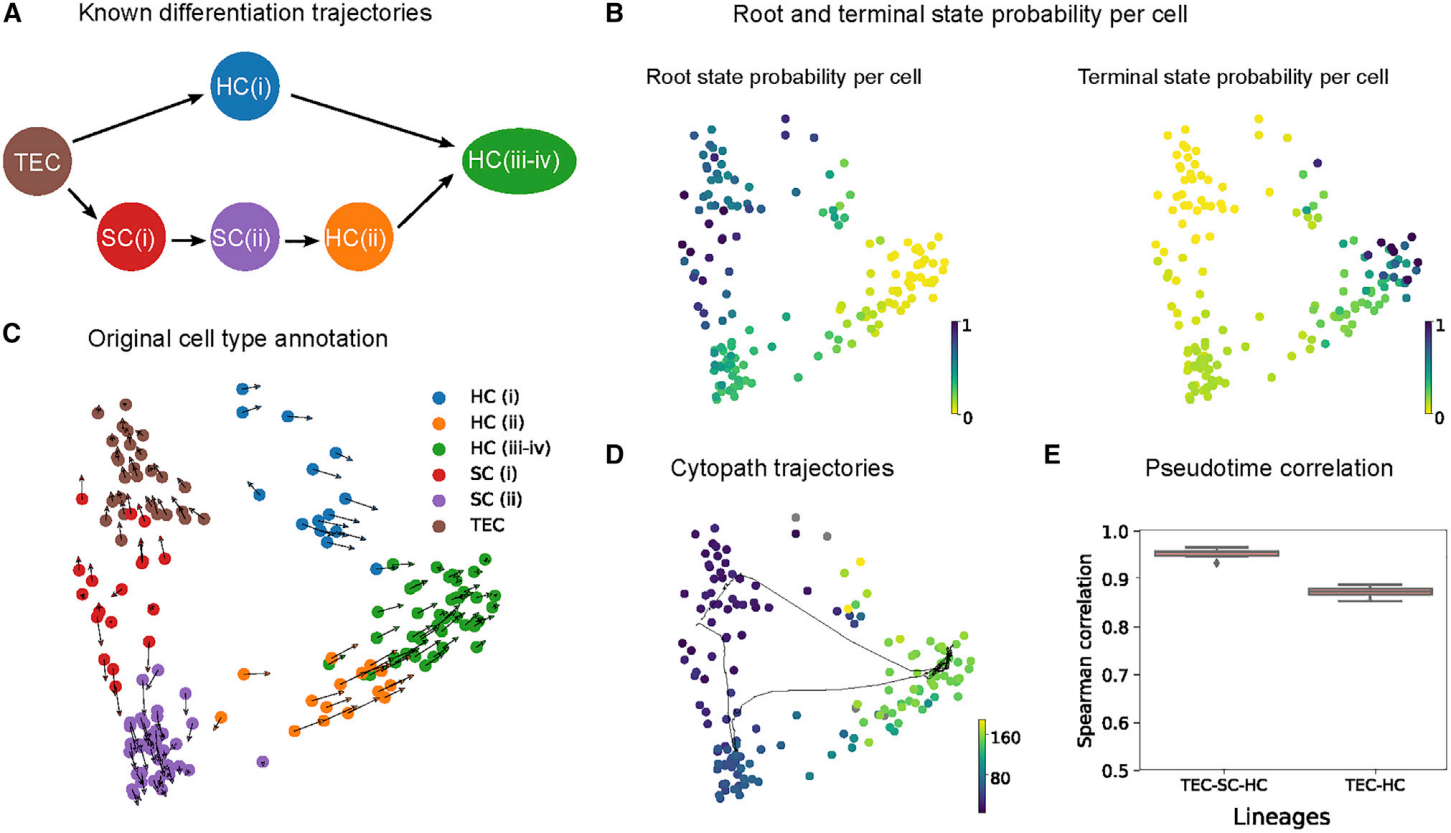
Reconstruction of convergent differentiation in the developing neonatal mouse inner ear (A) Known differentiation trajectories from Burns et al.^3^ (B) Probability estimated based on RNA velocity of a cell being a root state and terminal state, respectively. (C) RNA velocity overlayed on the PCA projection of neonatal mouse inner ear data, annotated with stages of differentiation. (D) Inferred trajectories and mean pseudotime by Cytopath. (E) Spearman correlations between known lineage ordering of cell types and pseudotime inferred by Cytopath (10 runs).

Burns et al.^3^ have shown that the development of hair cells (HCs) in the sensory epithelium of the utricle originates from transitional epithelial cells (TECs) via support cells (SC). This study also demonstrated a secondary differentiation path from TECs to HCs and put forward the existence of a transitional zone where cells can easily switch fate, resulting in two convergent differentiation trajectories^3^ (Figure 5A). This dataset presents a challenge for typical trajectory inference methods, given its relatively small size of 157 cells as well as a convergent differentiation topology that violates the topology-related assumptions of several methods.

Root and terminal state probability estimation using RNA velocity was used to select root and endpoints. Principal-component analysis (PCA) projection of the data was generated as indicated in the original study. Cytopath successfully models the two differentiation trajectories demonstrated in the study. The correlation between known cell type ordering and pseudotime inferred by Cytopath is robust for either lineage (Figures 5D and 5E).

Slingshot, VeTra, and Cellpath generate spurious trajectories that terminate at intermediate states (Figures S1D.2–S1D.4). None of these methods infer the convergent process. PAGA does not return an unbroken chain of cluster transitions with the default threshold (Figure S3D.4) Monocle3 requires a UMAP embedding; therefore, it was not benchmarked in this analysis.

### Cytopath pseudotime inference approximates the internal clock of cells

The difference between two expression states is sufficient to order the cells with respect to progression (difference in expression profiles), but without information about the rate of change of gene expression at any state, the pace of differentiation (i.e., the difference in expression profile relative to the internal clock) cannot be inferred. RNA velocity-based trajectory inference and pseudotime inference have the potential to resolve this drawback because RNA velocity provides an approximation of the rate of change of gene expression for each cell.

**Figure 6:**
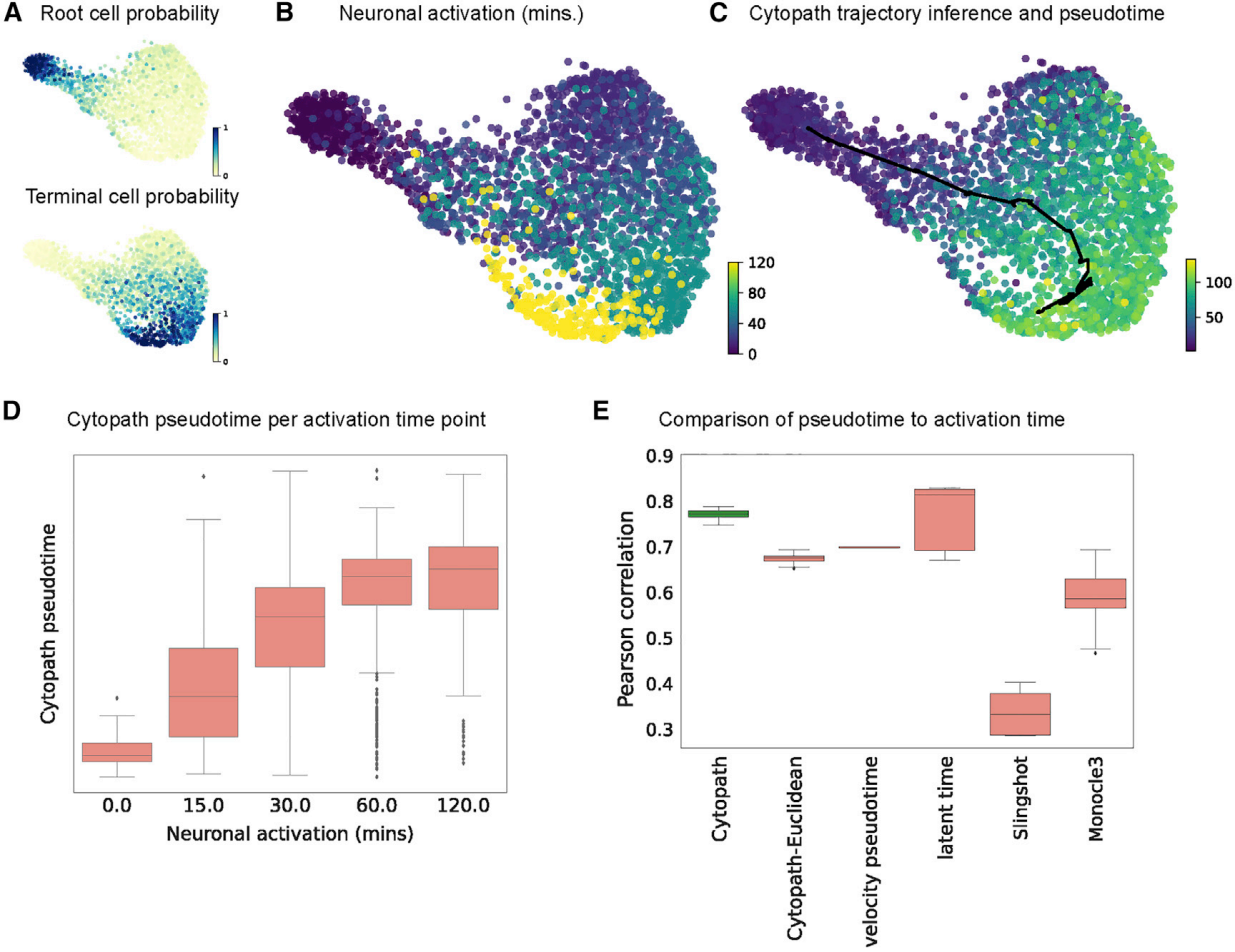
Reconstruction of ARG expression trajectory in mouse cortical neurons (A) Root and terminal state probability inferred using RNA velocity. (B) UMAP projection annotated with duration of stimulation for each cell. (C) UMAP projection annotated with trajectory and pseudotime inferred by Cytopath. (D) Cytopath pseudotime per cell with respect to stimulation duration. Note the monotonic relationship between median pseudotime and stimulation duration. (E) Pearson correlation between pseudotime inferred by Cytopath, non-velocity-based pseudotime inferred using Cytopath trajectory inference (Cytopath-Euclidean), and baseline methods.

Single-cell metabolically labeled new RNA tagging sequencing (scNT-seq) was developed as a means to experimentally measure the age of cells undergoing active transcription. To validate their method, Qiu et al.^13^ generated a dataset of mouse cortical neurons stimulated for durations ranging from 0–120 min (Figure 6B). The authors also identified a set of activity-regulated genes (ARGs) whose expression can be directly linked to the duration of stimulation. Unlike typical time-series scRNA-seq datasets, where the asynchronous expression of cells implies that the experimental time is decoupled from the internal clock, in the setting described above, the duration of stimulation is a representation of the biological process (internal clock) time with respect to the ARGs. We performed RNA velocity analysis followed by trajectory inference considering only ARG expression (Figures 6A and 6C). Pseudotime inferred by Cytopath has a monotonic relationship with stimulation time and high Pearson (linear) correlation (Figures 6C and 6D). To assess the specific relevance of RNA velocity in inferring a pseudotime that better approximates the internal clock, we computed a non-velocity-based pseudotime using the trajectory inferred previously by Cytopath (Cytopath-Euclidean pseudotime). This non-velocity pseudotime has lower median correlation over 10 runs than Cytopath pseudotime. Other velocity-based pseudotime estimates also have relatively higher correlation compared with non-velocity-based methods (Figure 6E).

In response to stimulation, neuronal cells undergo a relatively fast polarization phase and subsequently slowly return to a depolarized state that is similar to the root state in terms of expression but not rate of change of expression; i.e., RNA velocity. Cytopath-Euclidean pseudotime does not have a monotonic relationship with stimulation duration and places the 120-min group at a lower pseudotime. We observed the same pattern with Slingshot and Monocle3. However, this may partly be due to poor trajectory inference as well as non-velocity-based pseudotime inference. Surprisingly, latent time and velocity pseudotime, which are also RNA velocity-based pseudotime methods, also showed a similar pattern of lower pseudotime associated with the 120-min group of cells (see notebooks).

In the absence of an experimental measure of process time, it is difficult to conclusively explore the association of pseudotime with process time in other datasets presented in this paper. However, if we assume that RNA velocity is a good approximation of the transcriptional rate of change, then we find that pseudotime inferred using Cytopath outperforms non-velocity-based methods at approximating the real rate of change of transcription. The similarity of a cell’s velocity to each of its neighbors indicates the pace of coherent change of transcription in a region of transcriptional space. To quantify this property we define velocity cohesiveness (STAR Methods). High velocity cohesiveness indicates that the cell is present in a region of coherent and therefore rapid transcriptional change because the cell has a high transition probability to similarly oriented transition partners. Conversely, low velocity cohesiveness indicates that the rate of transcriptional change is low and that the cell transitions to its neighbors are less coherently directed. Because simulations generated by Cytopath are based on the aforementioned transitions, we expect that the pseudotime estimated by Cytopath better reflects the rate of real transcriptional change compared with a non-RNA velocity-based pseudotime that, by design, is forced to assume a uniform rate of transcription.

We tested this hypothesis by comparing pseudotime estimated using Slingshot and Cytopath for the pancreatic endocrinogenesis dataset. For each lineage, we estimated the relative rate of change of Slingshot pseudotime with respect to Cytopath pseudotime per cell. The high positive correlation between velocity cohesiveness and velocity magnitude indicates that, in regions with lower velocity magnitude, Slingshot has a lower rate of change in pseudotime compared with Cytopath and vice versa (Figures S4G and S4H). We define the simulation step density of a cell as the number of unique simulation steps visiting this cell. The negative correlation between simulation density per cell and velocity cohesiveness indicates an enrichment of transitions in regions of slower transcriptional change and vice versa (Figure S4I). The overall trajectory inferred from these simulations assigns a larger range of pseudotime values to regions with lower velocity cohesion because smaller changes in expression are associated with relatively larger passage of time compared with regions of higher velocity cohesiveness.

### Reconstruction of bifurcating differentiation of CD8^+^ T cells from scRNA-seq time series data

**Figure 7:**
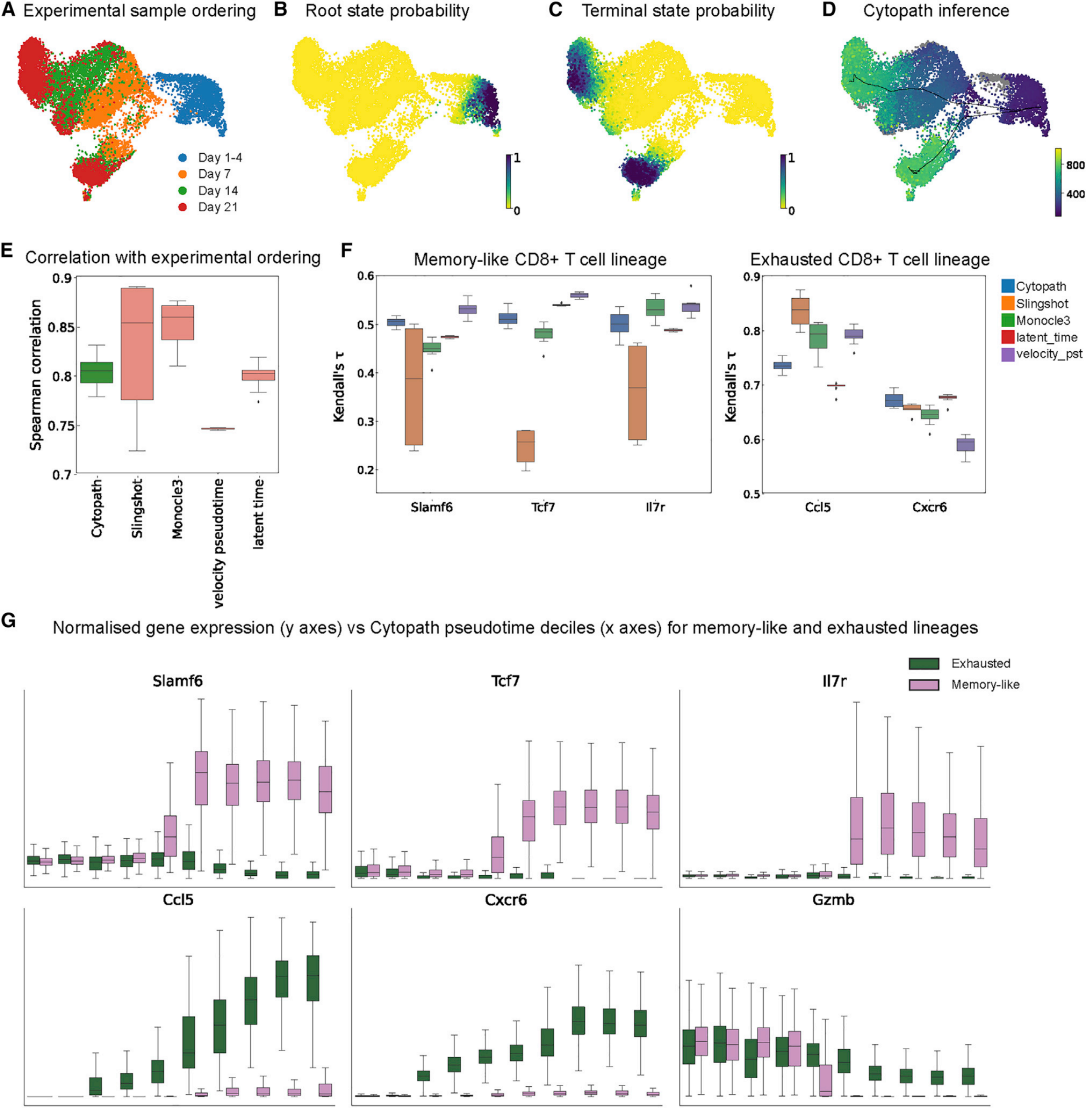
Reconstruction of bifurcating development of CD8^+^ T cells from scRNA-seq time series (A) Ordering of samples with respect to time of LCMV inoculation. (B and C) Probability estimated based on RNA velocity of the cell being (B) a root state (C) a terminal state. (D) Trajectories inferred and mean pseudotime per cell by Cytopath. (E) Correlation of pseudotime estimated by each method, with markers relevant to memory-like CD8^+^ T cell differentiation and exhausted CD8^+^ T cell differentiation, respectively. (F) Kendall’s tau correlation between marker expression and Cytopath pseudotime. (G) Normalized expression of key marker genes in each Cytopath pseudotime decile per lineage. Top row (*Slamf6*, *Tcf7*, and *Il7r*) markers are expected to be expressed in the memory-like lineage. *Ccl5* and *Cxcr6* are expected to be expressed only in the exhausted lineage. *Gzmb* is not expected to be differentially expressed between the two lineages.

We assessed the performance of Cytopath on an scRNA-seq time series dataset from CD8 T cell differentiation in chronic lymphocytic choriomenengitis virus [LCMV] infection.^4^ In this infection model system, CD8 T cells differentiate from early-activated cells into exhausted and memory-like cells over a period of 3 weeks. Samples were collected at eight experimental time points after infection with LCMV to cover all stages of the process and were sequenced in four batches (Figure 7A). Although these samples are heterogeneous snapshots of a spectrum of differentiation states at a particular time point, they provide an approximate development coordinate. Starting from a population of cells at an early-activated state, differentiation leads into the two distinct terminal states within 5 days of LCMV infection. This differentiation process is characterized by strong transcriptional changes and expression of different surface genes.

We identified the root and terminal states of the process using scvelo^8^ (Figures 7B and 7C). The terminal states were validated by expression levels of known marker genes.^4^ The exhausted terminal state showed high expression in co-inhibitory markers like CD39 (*Entpd1*), CD160 (*Cd160*), and PD-1 (*Pdcd1*). The memory-like terminal state had high expression of TCF1 (*Tcf7*) and interleukin-7R (IL-7R; *Il7r*). Trajectory inference with Cytopath resulted in two trajectories that led from a shared starting region to the two expected terminal regions. The two trajectories overlapped in the beginning of the process but then sharply diverged at a branchpoint (Figure 7D).

Comparing the pseudotime estimates from Cytopath with the discrete experimental time labels from the samples showed high agreement of the two. The experimental time labels, corresponding roughly to the developmental coordinate, were ordered correctly and with high Spearman correlation between pseudotime and time labels (Figure 7E). Unlike analysis of the neuronal activation dataset’s ARGs, the experimental time in this setting is not a precise representation of the internal clock of every cell. This is due to asynchronous activation of expression arising from cell-to-cell variation of antigen exposure times. Therefore, we only expect a correct ordering of experimental time labels with respect to median pseudotime per experimental sample and not a perfect correlation of pseudotime and experimental time label per cell.

Cytopath suggests a bifurcating trajectory model with trajectories originating at biologically relevant root states and terminating at either of the expected terminal states (Figure 7D). Slingshot also inferred trajectories to either terminus but generated a third spurious trajectory in the early-stage cell group that cannot be matched to any expected infection-induced differentiation trajectory (Figure S1F.2). VeTra and Cellpath infer multiple erroneous trajectories that do not initiate at the root state and estimate pseudotime that does not correspond to the differentiation process at all (Figures S1F.3, S1F.4, S2F.3, and S1F.4). Monocle3 reconstructs the global structure of the data but includes additional loops and branches in the exhaustion branch (Figure S3F.2).

We also assessed the correlation of pseudotime estimates with canonical gene expression markers of the memory-like (*Slamf6*, *Tcf7*, and *Il7r*) and exhaustion branch (*Ccl5* and *Cxcr8*). We observe that pseudotime inferred by Cytopath is highly correlated with lineage-specific markers (Figure 7F).

We then tested the validity of the average trajectories of Cytopath by the expression profiles of known lineage marker genes in the differentiation process. The chemokine receptor CXCR6 has been shown to mark exhausted T cells in chronic LCMV infection.^17^ The average expression of *Cxcr6* increases in the trajectory toward the exhausted cluster just prior to divergence of the two branches (Figure 7G), indicating that the paths are indeed governed by the exhaustion process. Conversely, T cell factor 1 (TCF1) and expression of its gene *Tcf7* are established markers of memory-like cells.^18^ Expression of this gene was increased in memory-like cells just after the cells started to diverge after the bifurcation point. Toward the memory-like terminal state at late time points, *Tcf7* expression is exclusive to the memory-like population. An additional observation is the high expression of *Gzmb* early in both branches that drops off toward later time points (Figure 7G). The expression of *Gzmb* is a shared feature of both branches and known to decrease in both branches as the infection progress and expression is low toward late timepoints.^19^

Cytopath is able to reconstruct biologically relevant differentiation trajectories from a long-term time series dataset in a more accurate and reproducible manner than widely used tools. We identified correct differentiation branches of CD8 T cells in chronic infection, demonstrated by correct ordering of the experimental time labels and expression levels of branch-specific gene expression markers. For this system, several phenotypic populations and characteristic markers have been described before, but the connecting differentiation trajectories of those populations are a subject of ongoing research.^20^^,^^21^^,^^22^^,^^23^ These studies provide evidence of branching in the development process, and only recently, in conjunction with simulation-based trajectory inference, has it been possible to resolve this event in more detail.^4^

## Discussion

Trajectory inference is a challenging task since scRNA seq data is noisy and - until recently - has been evaluated to achieve only static expression profile snapshots. Trajectory inference tools typically operate in low-dimensional embeddings, especially two-dimensional projections such as UMAP and t-SNE, possibly obfuscating complex trajectory topologies such as multifurcations and cycles because of more dominant sources of variation. Inclusion of directional transcriptional activity estimates from RNA velocity analyses is expected to achieve more precise and sensitive trajectory inference. With Cytopath, we present an approach that takes advantage of this information.

The transition probability matrix used to generate simulations is computed from high-dimensional gene expression and velocity profiles. Because Cytopath is based on transitions that use the full expression and velocity profiles of cells, it is less prone to projection artifacts distorting expression profile similarity. This approach specifically considers likely and discards unlikely transitions and therefore is able to identify, for instance, cyclic trajectories in an apparently diffusely populated and isotropic region of expression space (Figures 3 and 4). These hidden transcriptional patterns are made apparent by the simulation-based approach without any explicit supervision. Non-RNA velocity-based methods struggle to discriminate between cells corresponding to different stages or branches of cyclical and convergent processes, respectively, because the cells appear to be co-located in expression space. However, even RNA velocity-based methods^6^ do not readily present this information to the user, even when the pseudotemporal ordering or cell fate scoring estimated by these tools captures these patterns.

Cytopath analysis requires specification of the root and terminal regions. This requirement is met easily when the cellular process is sufficiently well characterized up to the level of *a priori* definition of these regions. However, even when this is not the case, Cytopath can detect and utilize tentative root and terminal states from the cell-to-cell transition probability matrix using scvelo.^8^ We found pseudotime inferred by Cytopath to be robust to root and terminal probability thresholds within a range of [0.85, 1] (data not shown). Simulations generated using Cytopath can also aid identification of terminal cell types. Compared with the absorbing Markov process-based inference of terminal states implemented in velocyto and scvelo, this approach appears to highlight more terminal states in the pancreatic dataset (Figure S5B3). Intermediate quasi-stationary cell states induced, for instance, by a switch of transcriptional programs appear to be highlighted by this procedure, as indicated by the switch from the cell cycle to islet cell differentiation in the pancreatic endocrinogenesis study.

Although Cytopath is primarily a trajectory inference tool, we leverage the alignment-based association of cells to inferred trajectories to generate additional results that overlap in functionality with other RNA velocity-based tools. For instance, Cytopath can also be used to predict cell fate. Differentiation potential of cells estimated as the entropy of cell fate probability across the terminal states can be used to investigate branching events (Figure S6).

Other RNA velocity-based trajectory inference tools, VeTra and Cellpath, appear to perform significantly worse than non-velocity-based methods used as benchmarks in this study (Figures S1, S2, and S3). We assume that the reason for the difficulty to recapitulate trajectories could be their built-in lack to guide trajectory inference by separately providing root and terminal states. This appears to be a contrived problem because biological knowledge regarding the identity and role of cells is typically available or, as discussed before, can be estimated separately. Ignoring this information seems to make trajectory inference unnecessarily difficult and could be the reason why Cytopath, as well as Slingshot and Monocle3, perform better than VeTra and Cellpath. Regarding trajectory inference with Cytopath, we find that automatic selection of root and terminal states tends to match biologically relevant root and terminal states, with the strong exception of the pancreatic endocrinogenesis dataset. In general, we recommend that root and terminal state selection should be done by synthesizing all available sources of information, including application-relevant cell type marker expression profiles, analytically derived probabilities based on RNA velocity, and simulations using Cytopath.

PAGA^15^ is another popular tool that can include RNA velocity to infer directed connectivity between clusters of an scRNA-seq dataset. Velocity pseudotime allows directed edges to be inferred using PAGA, but an unbroken sequence of connections is not guaranteed (Figures S3A.4–S3F.4). The coarse graph approach has a few disadvantages compared with trajectory inference methods. Dedicated trajectory inference methods such as Cytopath, Slingshot, and Monocle3 can model gradual divergence of lineages. Cell fate scoring estimated by Cytopath constitutes fuzzy assignment of cells to multiple lineages. These methods also support relatively diffuse regions of branching. In contrast, PAGA considers cells in a cluster to be homogeneous with respect to lineage assignment; therefore, branching can only be defined at the cell cluster level.

Addition of RNA velocity is expected to allow pseudotime inference that is a better representation of the internal clock of the cell that corresponds to the pace of differentiation. We show that pseudotime inferred by Cytopath has a monotonic relationship with the process time. We show three points of evidence in this study. The first is the ability to partition cells in the G1 phase of the cell cycling dataset into late- and early-stage cells. From the perspective of gene expression profiles, these cells are co-clustered and appear as a single cell type. However, the RNA velocity-based cell-to-trajectory alignment procedure implemented in Cytopath assigns these cells to an early trajectory step corresponding to G1-S phase transition or a late stage indicating G1-to-G1 checkpoint transition. The biological relevance of this partitioning can be validated by the significant difference in gene expression of a selected set of cell cycle marker genes (Figure 3A). Second, we investigate in more detail the correlation of pseudotime inferred by Cytopath with stimulation duration for the neuronal activation dataset. By restricting the analysis to ARGs whose expression is triggered in response to stimulation and, hence, synchronized per cell, we can consider the experimental time ordering to be coupled to the process time in this dataset (Figure 6). Third, we examine the relationship between velocity magnitude and rate of change of pseudotime. Intuitively, regions with high velocity magnitude are expected to have relatively larger change of expression with respect to the internal clock of cells and vice versa. We show that this relationship is better modeled by Cytopath than non-velocity-based methods (Figures S4G–S4I). Aforementioned results suggest that pseudotime estimated by Cytopath is an improvement on approximating of real rate of change of gene expression.

Cytopath considers generic properties of scRNA-seq datasets, such as the total number of cells and number of inferred root and terminal states, to initialize the hyperparameters of the trajectory inference process. This selection is done with the objective of computational efficiency as well as robust detection of trajectories. All analyses presented in this study utilized the default automatic hyperparameter selection approach, but users still have the option of performing manual tuning.

We expect simulation-based trajectory inference approaches like Cytopath to enable sufficiently precise and unambiguous trajectory inference to achieve testable hypotheses to identify drivers and derive mathematical models of complex differentiation processes.

### Limitations of the study

In certain datasets, RNA velocity estimation could be unrepresentative of the true transcriptional dynamics. These issues arise from the simplifying assumptions of time-invariant rates of transcription, splicing, and degradation as well as the assumption of each gene operating under a single regulatory regime. Although these issues typically lead to erroneous inference of dynamics for only a few genes, it is possible for the overall process to be incorrectly modeled when the dynamics of a high proportion of genes are modeled incorrectly. Particular scenarios where RNA velocity estimation fails to recapitulate known dynamics have been explored by Bergen et al.^24^

In the context of trajectory inference with Cytopath, the overall structure and directionality is inferred using the transition probability matrix, which, in turn, represents the aggregate behavior of RNA velocity of all genes included in the analysis. We do not expect that trajectory inference with Cytopath is meaningful when the underlying RNA velocity estimation itself is faulty.

Specifically, incorrect RNA velocity estimation has an effect on inference of root and terminal cell states (Figure S7). For the erythroid gastrulation dataset, a spurious set of root points is inferred toward the terminus of the expected process, and the endpoints are spuriously inferred in the middle of the expected ordering of cell types (Figure S7B). With the parameterization used by Bergen et al.,^24^ we generated a simulated dataset (scvelo simulation function) consisting of features with time-dependent degradation rates (Figures S7E and S7F) and observed a similarly spurious inference of root and terminal states. RNA velocity approaches are typically further challenged when the dataset is composed of mature, terminally differentiated cell types. In a dataset composed of cells representing hepatocyte zonation, we observe a clear directionality inferred using RNA velocity even though no expression dynamics are expected. The root cell probability estimation appears to be fuzzy, and the root cell probability is distributed across the dataset with no discernible pattern (Figures S7C and S7D). We do not recommend performing trajectory inference with Cytopath when the root and terminal cell states are not clearly identifiable. Although not applicable in every scenario when independent sources of information regarding the biological identity of cells are available, such as expression of validated expression markers, we recommend that users verify the plausibility of root and terminal states before proceeding with trajectory inference.

## STAR★Methods

### Key resources table

**Table:** 

REAGENT or RESOURCE	SOURCE	IDENTIFIER
**Deposited data**		

Dentate Gyrus	Hochgerner et al.^25^	GEO:GSE95753
Cell cycle	Mahdessian et al.^5^	GEO:GSE146773
Pancreatic endocrinogenesis	Bastidas-Ponce et al.^2^	GEO:GSE132188
Neonatal mouse inner ear	Burns et al.^3^	GEO:GSE71982
CD8 development	Cerletti et al.^4^	ENA:PRJEB43201
Neuronal activation	Qiu et al.^13^	GEO:GSE141851
Erythroid gastrulation	Pijuan-Sala et al.^26^	ArrayExpress:E-MTAB-6970
Hepatocyte zonation	MacParland et al.^27^	GEO:GSE115469

**Software and algorithms**		

Cytopath v0.1.8.post1	This manuscript	https://github.com/aron0093/cytopath https://doi.org/10.5281/zenodo.7278035
Slingshot v2.2.0	Street et al.^9^	https://github.com/kstreet13/slingshot
Monocle3 v1.0.0	Cao et al.^16^	https://github.com/cole-trapnell-lab/monocle3
VeTra commit 63e638c1d60c9faa46f74 e8ce5a226c8d9f5c40e	Weng et al.^11^	https://github.com/wgzgithub/VeTra
Cellpath v0.2.dev0	Zhang et al.^12^	https://github.com/PeterZZQ/CellPath
Cellrank v1.5.1	Lange et al.^6^	https://github.com/theislab/cellrank
scvelo v0.2.4	Bergen et al.^24^	https://github.com/theislab/scvelo
dynverse/ti_angle:v0.9.9.02	Saelens et al.^14^	https://github.com/dynverse/dynmethods/blob/master/R/ti_angle.R
dynverse/ti_recat:v0.9.9.01	Saelens et al.^14^	https://github.com/dynverse/dynmethods/blob/master/R/ti_recat.R
Python v3.8.6	Python Software Foundation.	https://www.python.org/
R v4.1.2	R Core Team.^28^	https://www.r-project.org/

**Other**		

Dentate Gyrus	La Manno et al.^1^	https://github.com/velocyto-team/velocyto-notebooks/blob/master/python/DentateGyrus.ipynb
Cell cycle	Mahdessian et al.^5^	https://github.com/CellProfiling/SingleCellProteogenomics/
Pancreatic endocrinogenesis	Bergen et al.^24^	https://scvelo.readthedocs.io/scvelo.datasets.pancreas/#scvelo.datasets.pancreas
Erythroid gastrulation	Bergen et al.^24^	https://scvelo.readthedocs.io/scvelo.datasets.gastrulation_erythroid/#scvelo.datasets.gastrulation_erythroide
Hepatocyte zonation	Pijuan-Sala et al.^26^	https://github.com/BaderLab/HumanLiver
Simulation	Bergen et al.^24^	https://scvelo.readthedocs.io/scvelo.datasets.simulation/#scvelo.datasets.simulation

### Resource availability

#### Lead contact

Further information and requests for resources and reagents should be directed to and will be fulfilled by the lead contact, Manfred Claassen (manfred.claassen@med.uni-tuebingen.de).

#### Materials availability

This study did not generate new unique reagents.

### Method details

#### Trajectory inference with cytopath

##### Cell clustering

Any grouping of cell states, such as clustering from widely used community detection algorithms or cell type annotations generated by domain experts can be provided as input to Cytopath.

For the set of all cells C and the set of all clusters S, the clustering is $f^{s}:C\to S$ where |S|<|C|.

By default Cytopath will perform clustering of cells using Louvain via *scvelo*.^8^

##### Stationary state selection

Root $\left(P_{r}\right)$ and terminal $\left(P_{t}\right)$ state probabilities are used to determine the stationary states as described previously.^1^^,^^8^ By default, a threshold of 0.99 is used.

The set of root cells $C^{r}$ is defined as $\left\{c\in C:P_{r}\left(c\right)\geq 0.99\right\}$. Similarly, the set of all terminal cells $C^{t}$ is defined as $\left\{c\in C:P_{t}\left(c\right)\geq 0.99\right\}$. Terminal regions $S^{t}$ are defined as $\left\{f^{s}\left(c\right)\in S:c\in C^{t}\right\}$.

When prior biological knowledge regarding the data exists, users can also manually specify root and terminal cell states or designate entire clusters as root or terminal regions.

##### Simulations

Simulations are initialized at random cell states selected uniformly within the defined root cells and consist of a fixed number of cell to cell transitions.

At each step, a single transition from the current cell state is realised based on the cell to cell transition probability matrix P . Each row of the matrix contains the probability of transition from the current state (row index) to another cell in the dataset (column index). The cell state $c_{ij}$ at step i of simulation j is selected randomly according to P from the nearest neighbors of $c_{\left(i-1\right)j}$.

Let F be the cummulative probability distribution of P . A value κ is sampled from a uniform distribution over [0, 1) and,

$$c_{ij}=argmin_{c\in C}\left(F\left(c/c_{\left(i-1\right)j}\right)-\kappa \right)∋F\left(c/c_{\left(i-1\right)j}\right)\geq \kappa$$

##### Technical parameter selection

Under default settings, the number of simulation steps and minimum number of simulations to be generated are automatically adjusted based on the size of the dataset and number of terminal regions. The purpose of this adjustment is to make the sampling process computationally efficient. The scaling parameters were determined by empirical testing.

The number of simulations steps $i_{max}$ is initialised as $\left[5*\;log_{10}\;\left(\left|C\right|\right)\right]$ This represents an increase of five simulation steps per order of magnitude increase in the size of the dataset. The minimum number of unique simulations to be generated per terminal region m is selected as $\left[500*\;log_{10}\;\left(\left|C\right|\right)\right]$.

Subsequently the number of simulation steps and number of simulations to be sampled are adjusted during the sampling process in an iterative fashion based on the proportion of simulations that terminate at terminal regions in the previous iteration, until the minimum number of unique simulations per terminal region have been generated.

Let J be the set of all simulations generated in an iteration and $J^{t}$ be the set of simulations terminating in terminal regions. If $\left|J^{t}\right|\leq 0.1*\left|J\right|$ then the number of simulation steps is doubled.

Let $J_{lag}^{t}$ be the set of simulations terminating at the terminal region with least number of simulations, $\min_{s\in S^{t}}\left|J_{s}^{t}\right|$. If $\left|J_{lag}^{t}\right|<0.6*m$ then the number of simulation steps are incremented by $i_{max}*\left(m/max\left(i_{max},\left|J_{lag}^{t}\right|\right)\right)$.

If the two conditions above are met and the minimum number of unique simulations per terminal region are not obtained for any terminal region then more samples are generated until $\left|J_{lag}^{t}\right|==m$.

##### Trajectory inference

Simulations that terminate within terminal regions are considered for trajectory inference. Trajectory inference is performed by first clustering the simulations and then aligning them using Dynamic Time Warping, which is an algorithm that allows alignment of temporal sequences with a common origin and terminus that possibly have different rates of progression.

For any two simulations $A=\left\{c_{0a}\ldots c_{ia}\right\}$ and $B=\left\{c_{0b}\ldots c_{ib}\right\}$, the Euclidean Hausdorff distance H(A,B) is defined as,

$$H\left(A,B\right)=\;\max\left(\max_{c_{a}\in A}\left(\min_{c_{b}\in B}d\left(c_{a},c_{b}\right)\right),\max_{c_{b}\in B}\left(\min_{c_{a}\in A}d\left(c_{b},c_{a}\right)\right)\right)$$

where d is Euclidean distance. Simulations terminating within a single terminal region $J_{s}^{t}$ for $s\in S^{t}$, are clustered using Louvain based on Euclidean Hausdorff distance.

Each cluster of simulations is then aligned in a greedy-pairwise fashion using the *fastdtw* python package to generate a single ensemble sequence per cluster which we refer to as a sub-trajectory.^29^

The mean value of coordinates of cells at each step of the aligned simulations is the coordinate of the (sub-) trajectory at that step. Subsequently, a second round of clustering and alignment is performed, using the sub-trajectories from the first round to produce trajectories that are reported by Cytopath. By default the number of expected trajectories per terminal region is not specified allowing from unbiased inference of multiple trajectories per terminal region. However, users have the option to manually enforce the number of trajectories to infer per terminal region.

##### Identification of compositional clusters

For each trajectory compositional clusters are identified from the set of clusters provided in the step *cell clustering*. For each step i of the trajectory, its neighborhood $M_{i}$ in PCA space is recovered with a K-dimensional tree search.^30^ Cell clusters with a representation larger than a threshold frequency (Default:0.3) for at least one $M_{i}$ are considered compositional clusters of that trajectory.

##### Alignment score

After the trajectory coordinates have been inferred, the cell-to-trajectory association is computed. Trajectories inferred by Cytopath are segmented and cells in the compositional clusters of a trajectory are aligned to the segments of the trajectory.

For a cell with neighbors K, its alignment score to step i of a trajectory is the maximum of two scores. The score with respect to the trajectory segment b from steps i−1 to i, $\xi_{i}^{b}$ is calculated as,

$${\text{ξ}}_{i}^{b}=\frac{1}{\left|K\right|}\sum_{k}^{K}cos\left({\text{η}}_{k}^{b}\right)\cdot exp\left({\text{γ}}_{k}\right)$$

where η is the cosine angle between the section of the trajectory and all possible transition partners k of the cell. γ is the cosine similarity between the velocity vector of the cell with the distance vector between the cell and its neighbors.

The score with respect to the trajectory segment f from steps i to i+1, $\xi_{i}^{f}$ is calculated similarly,

$${\text{ξ}}_{i}^{f}=\frac{1}{\left|K\right|}\sum_{k}^{K}cos\left({\text{η}}_{k}^{f}\right)\cdot exp\left({\text{γ}}_{k}\right)$$

The alignment score is an extension of the transition probability estimation implemented in *scvelo* by weighting each transition of a cell with respect to its alignment to a trajectory. The score is used for assessing the position of a cell with respect to a trajectory (pseudotime) and subsequently to compute a fate score with respect to multiple lineages. The alignment score $p_{i}$ of the cell with respect to step i is $max\left({\text{ξ}}_{i}^{b},{\text{ξ}}_{i}^{f}\right)$.

For each cell the final alignment score *p* with respect to a particular trajectory is an average of its alignment scores to multiple step segments of the trajectory. By default, it is the mean, however other averages can also be used.

##### Cell fate score

For each cell its alignment score, relative to multiple trajectories is the cell fate score. Cell fate score $f_{traj}$ for cell with respect to a trajectory is

$$f_{traj}=\frac{p_{traj}}{\sum_{trajectories}p_{traj}}$$

##### Differentiation potential

For each cell the entropy of its cell fate distribution over all terminal states of the dataset was estimated using *scipy.stats.entropy* function. The values were scaled to range [0, 1] over the dataset.

##### Pseudotime estimation

For each trajectory cells that have an alignment score greater than zero and also belong to a compositional cluster of the trajectory are assigned a pseudotime value with respect to the trajectory. Since a cell can align to multiple steps within a trajectory, the mean step value of a cell weighted by alignment score is taken as its pseudotime value, for each trajectory. Optionally, other averages can also be used.

### Quantification and statistical analysis

#### Evaluating dynamical properties of cytopath pseudotime

##### Terminal state identification using undirected simulations

For each dataset, five thousand simulations were initialized at randomly chosen cells from the dataset and sampling was performed for 30 steps. The log of total count of simulations, from all the clusters, terminating at each cell, rescaled to range [0,1] is reported as propensity for constituting either a terminal state or an intermediate state representing a switch in transcriptional programme.

##### Cytopath-Euclidean pseudotime

Cytopath-Euclidean pseudotime was computed by considering all the cell-to-trajectory alignments inferred by Cytopath. The cell was assigned to the trajectory segment with minimum Euclidean distance between the cell and the segment in PCA space.

##### Velocity cohesiveness

Cosine similarity of the distance vector between a cell to its transition partners, and the cell’s velocity vector, per cell is stored as the velocity graph. Velocity cohesiveness of cells are the mean values of each row of this matrix.

##### Pseudotime comparison

Cells were ordered by Cytopath pseudotime. For each cell a window of 50 cells centered on the cell was considered. For each window a linear model was fit with respect to Slingshot and Cytopath pseudotime using *scipy.stats.linregress* function. Rate of change of Slingshot pseudotime vs Cytopath pseudotime was assessed by estimating the slope.

##### Simulation step density

Simulation step density per cell is the log of the count of simulation steps that are a transition to the cell.

##### Runtime analysis

*process_time* function from the *time* package was used to record the time taken for each trajectory inference procedure.

#### Comparison of trajectory inference approaches

##### Parameter settings

Default settings were used for all methods except VeTra and Cellpath. Recommended settings based on information published by the authors were used for VeTra and Cellpath. Cytopath was run using default parameters for all datasets. The same root and terminal states used for trajectory inference with Cytopath were provided to other methods. Figure S5 shows the root and terminal state probabilities estimated using *scvelo* for each dataset.

##### Pseudotime comparison

Spearman correlation of the pseudotime values generated by each method with the cell type cluster ordering for each biological lineage was used to compare the performance of the methods. Kendall’s tau was used to assess the correlation of marker expression with the estimated pseudotime. For each dataset and method, analysed with the correlation analysis described above, the analysis was performed on the dataset with ten independent initialisations of the entire processing pipeline.

#### RNA velocity analysis

Pre-processed data was used wherever available. Subsequent analysis was performed with *scvelo* using standard workflow.

##### Dentate gyrus

Prepossessing was performed as indicated by the authors of the original study using code published by La Manno et al.^1^

##### Neonatal mouse inner ear

Raw sequencing data was downloaded from the NCBI GEO database under accession code GSE71982. Quality control including read filtering and adaptor trimming was performed using *fastp*.^31^ Reads were aligned to the GRCm39 mouse genome assembly using *STAR version=2.7.8a-2021- 04-2*.^32^ Spliced and unspliced counts were estimated using the *velocyto run-smartseq2* command following the recommendation of the developers.

##### CD8 development

Read counts were realigned and sorted for spliced and unspliced counts using the analysis pipeline from velocyto.^1^ Other contaminating cell types were removed from the dataset based on outliers in diffusion components.^4^

##### Hepatocyte zonation

Data corresponding to clusters Hep 1, 2 and 4 from patient 3 was used to perform the analysis.^27^

Read counts were realigned and sorted for spliced and unspliced counts using the analysis pipeline from velocyto.^1^

### Additional resources

Trajectory inference analysis with Cytopath including pre-processing and velocity analysis for each dataset presented in this paper can be found at https://github.com/aron0093/cytopath-notebooks. Download links for *anndata* objects for each dataset are also available in the corresponding notebook.

Documentation including installation instructions can be obtained at https://cytopath.readthedocs.io/.

## Data Availability

• Raw data for all datasets analyzed in this study are accessible on public repositories. The accession numbers are listed in the key resources table. Links to processed data, if available, have also been provided. • Cytopath has been implemented as a python package and can be found at the following GitHub repository (https://github.com/aron0093/cytopath) and at https://doi.org/10.5281/zenodo.7278035. • Cytopath is also available for installation via PyPI using the command ‘pip install cytopath’. • Any additional information required to reanalyze the data reported in this paper is available from the lead contact upon request.

## Supplemental information

Document S1. Figures S1–S7 and Table S1

Document S2. Article plus supplemental information
