# Supplementary material for: Simulation-based inference of differentiation trajectories from RNA velocity fields
Source: Cell Rep Methods. 2022 Dec 19;2(12):100359. doi: 10.1016/j.crmeth.2022.100359 (PMC9795361; doi:10.1016/j.crmeth.2022.100359)
Supplement: Document S1. Figures S1–S7 and Table S1 [file mmc1.pdf]

Cell Reports Methods, Volume *2*

## Supplemental information

# Simulation-based inference of differentiation trajectories from RNA velocity fields

**Revant Gupta, Dario Cerletti, Gilles Gut, Annette Oxenius, and Manfred Claassen**

**SUPPLEMENTAL TABLES & FIGURES**

| | Pseudotime inference | Trajectory coordinates | Branch assignment | Cell fate scoring | Coarse cell type graph | Optional root state input | Optional terminal state input | RNA velocity based |
|---|---|---|---|---|---|---|---|---|
| Cytopath | X | X | X | X | X | X | X | X |
| Slingshot | X | X | X | | X | X | X | |
| Monocle3 | X | X | | | | X | | |
| VeTra | X | | X | | | | | X |
| Cellpath | X | X | X | | | | | X |
| Cellrank | | | | X | | | | X |
| Scvelo-latent time | X | | | | | X | X | X |
| Directed PAGA | | | | | X | X | X | X |

**Table S1** Features of tools modelling differentiation processes from sc-RNA seq. data. Related to STAR Methods.

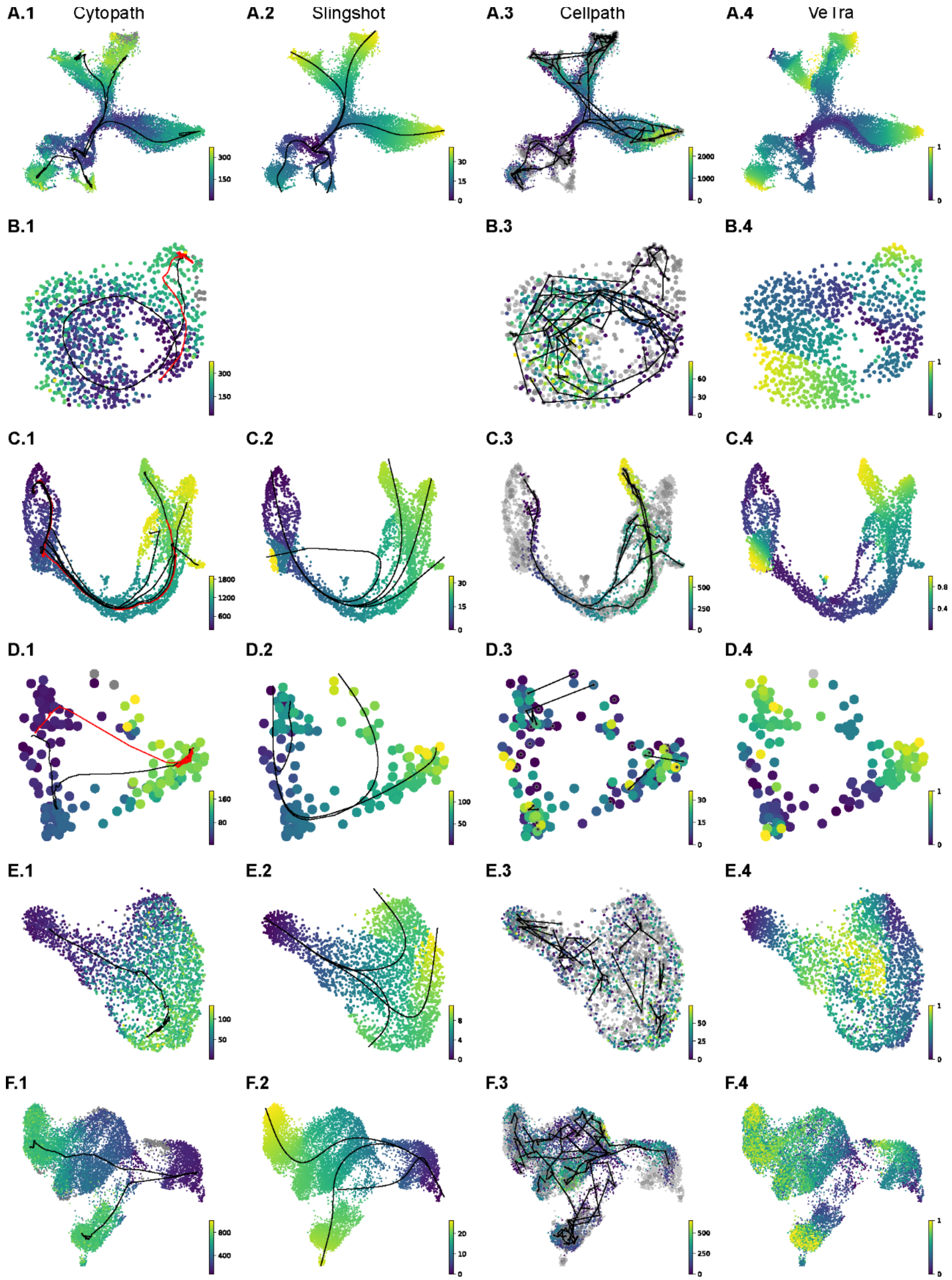


**Figure S1. Average pseudotime and trajectories inferred by trajectory inference methods that assign cells to lineages and therefore compute a trajectory specific pseudotime. Related to STAR Methods.** (A-F) Average pseudotime by Cytopath, Slingshot, Cellpath and VeTra for (A) Dentate Gyrus, (B) cell cycle, (C) pancreatic endocrinogenesis, (D) mouse inner ear (E) neuronal activation and (F) CD8+ T cell exhaustion datasets. Expected behaviour is that all terminal cell states have the highest pseudotime and root states have the lowest pseudotime. Erroneous trajectories initialised at terminal or intermediate states will lead to a patch-like appearance.

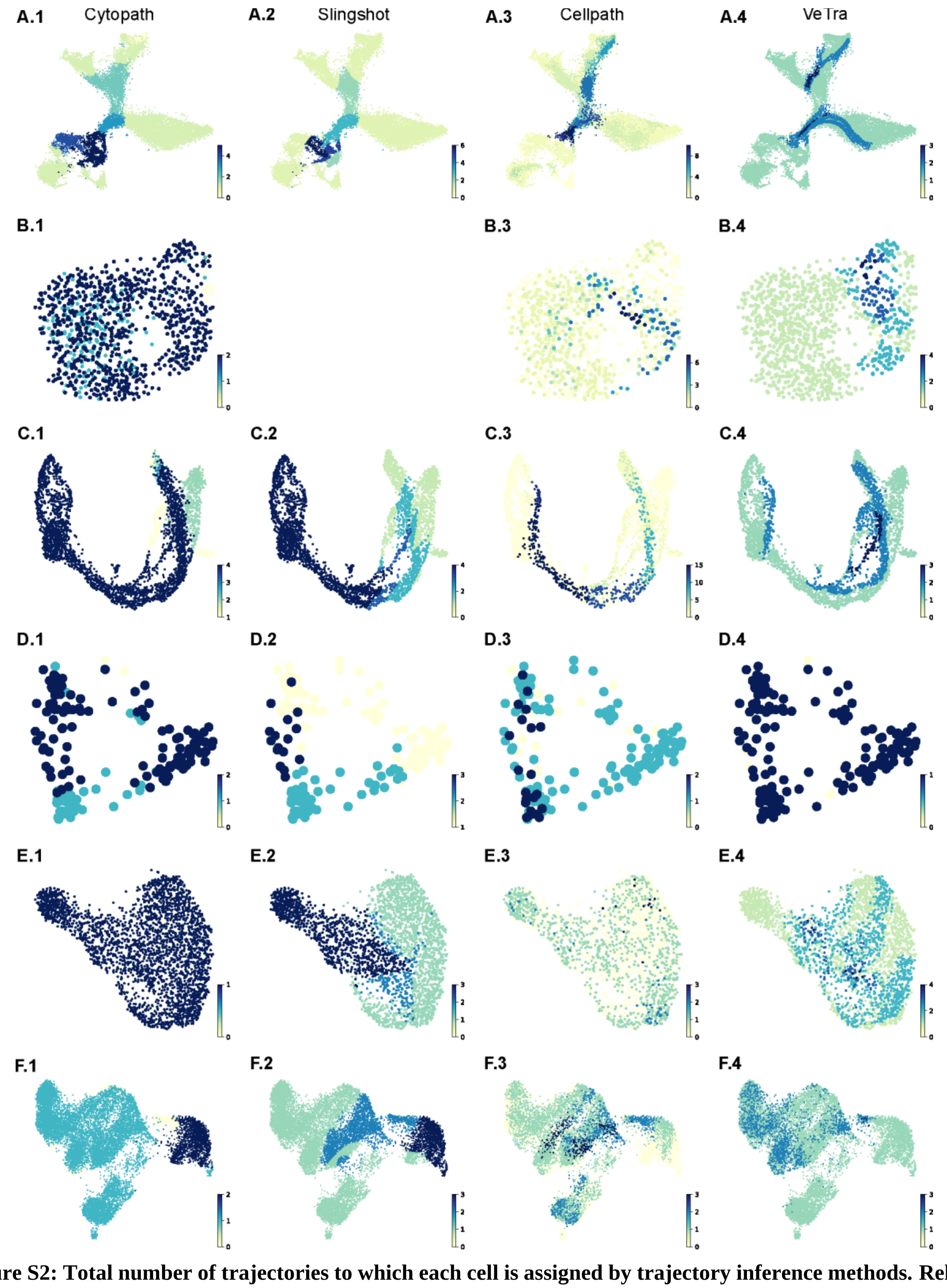


**Figure S2: Total number of trajectories to which each cell is assigned by trajectory inference methods. Related to STAR Methods.** (A-F) Count of trajectories to which each cell is assigned by Cytopath, Slingshot, Cellpath and VeTra for (A) Dentate Gyrus, (B) cell cycle, (C) pancreatic endocrinogenesis, (D) mouse inner ear (E) neuronal activation and (F) CD8+ T cell exhaustion datasets. Expected behaviour is that terminal state cells are assigned to only one trajectory in a multi-terminal state dataset. Cells at the root are typically assigned to all trajectories with each branching event lowering the count. Zero count indicates that the cell was missed by the trajectory inference process entirely.

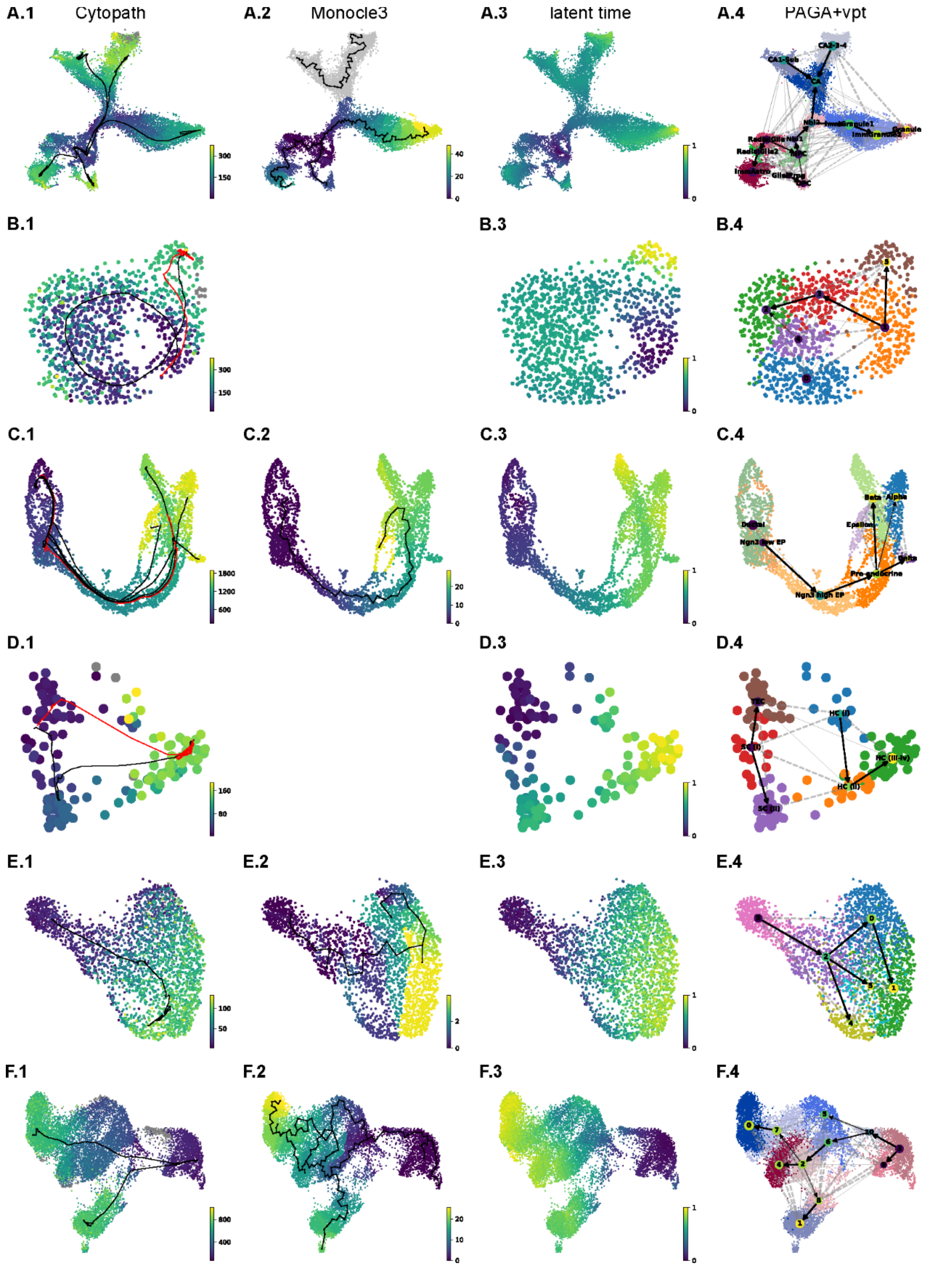


**Figure S3: Pseudotime and/or trajectories/graph inferred by methods that compute a global pseudotime per dataset and do not assign cells to lineages. Related to STAR Methods.** Cytopath pseudotime and trajectories are provided as reference. (A-F) Pseudotime and trajectories/graph by Cytopath, Monocle3, latent time (scvelo) and PAGA with directionality imparted by velocity pseudotime (vpt). (A) Dentate Gyrus, (B) cell cycle, (C) pancreatic endocrinogenesis, (D) mouse inner ear (E) neuronal activation and (F) CD8+ T cell exhaustion datasets.

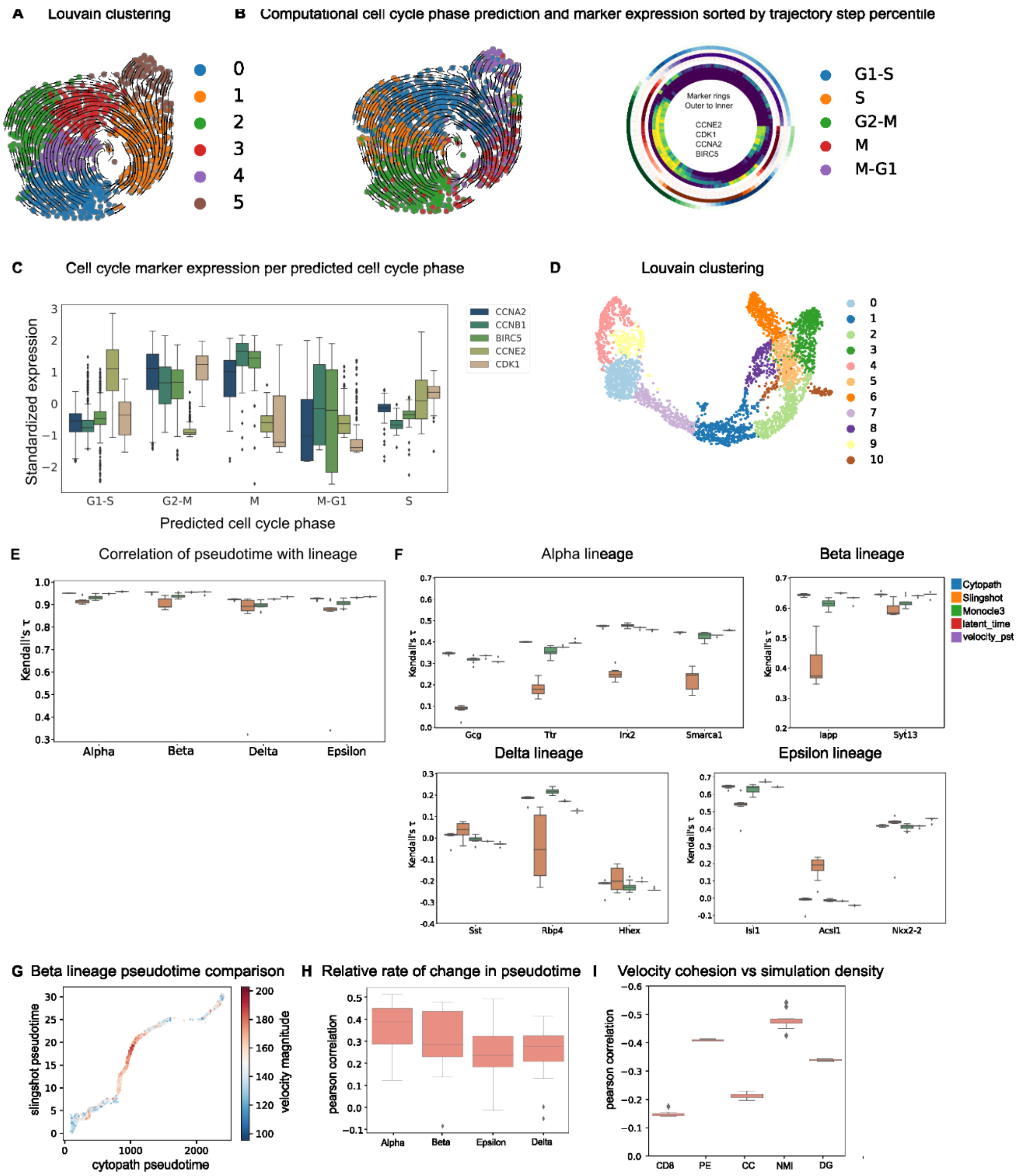


**Figure S4: Supplementary analysis. Related to Figures 3, 4 and 6.** (A) Louvain clustering of the cell cycle dataset. Cluster 5 corresponds to G1-checkpoint cells. (B) RNA velocity stream plot overlayed on the UMAP projection, annotated with cell cycle phase predicted computationally using expression data. Considering all cell-to-trajectory alignments binned into percentiles, the radial heatmap shows cell cycle phase fraction (outer set of rings) and marker expression (inner set of rings) sorted by trajectory step. The directionality of the radial heatmap is clockwise with the origin at zero degrees. (C) Expression of cell cycle markers for each predicted cell cycle phase. (D) UMAP projection of the pancreatic endocrinogenesis dataset annotated with Louvain clustering. (E) Spearman correlation of pseudotime values assigned by each method to the known ordering of cell types per trajectory. (F) Correlation of pseudotime estimated by each method with markers relevant to the trajectories inferred for each terminal cell type. (G) Slingshot vs Cytopath pseudotime for beta lineage annotated with magnitude of RNA velocity per cell. (H) Relative rate of change of Slingshot pseudotime vs Cytopath pseudotime suggests that Cytopath pseudotime better represents transcriptional dynamics. (I) Correlation of simulation step density for trajectories inferred by Cytopath demonstrate lower density of steps in regions of directed velocity i.e. the trajectories are modelled as transiting faster through regions of consistent directionality and vice-versa.

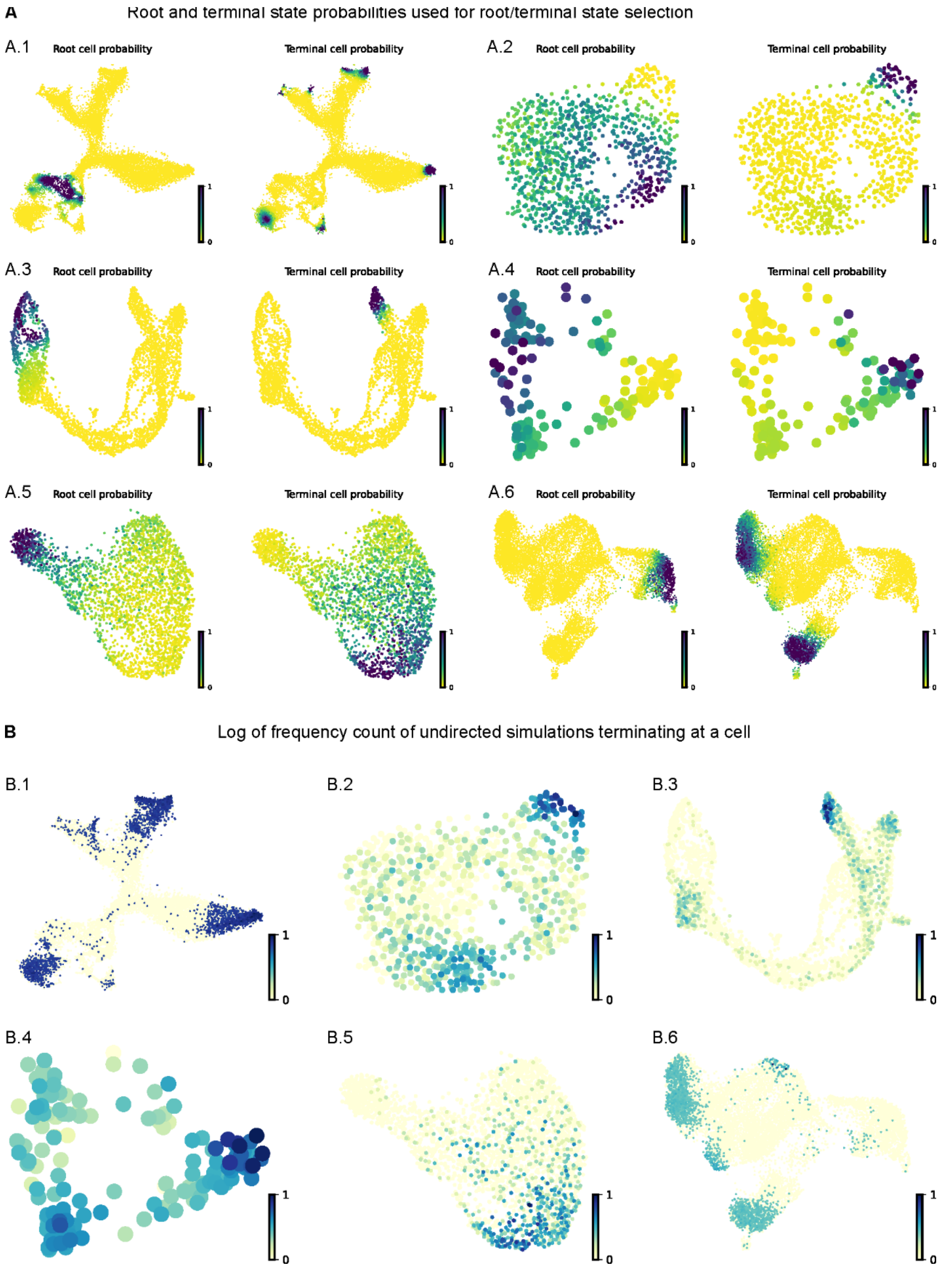


**Figure S5: Root and terminal state probability estimation based on RNA velocity. Related to STAR Methods.** (A) Root and terminal state probability of cells estimated using scvelo for the (A.1) Dentate gyrus, (A.2) cell cycle, (A.3) pancreatic endocrinogenesis, (A.4) neonatal mouse inner ear, (A.5) neuronal activation and (A.6) CD8 T cell development datasets. (B) Log of frequency count of undirected simulations (initialised at random cell states) terminating at a cell scaled to range [0,1] for (B.1) Dentate gyrus, (B.2) cell cycle, (B.3) Pancreatic endocrinogenesis, (B.4) neonatal mouse inner ear, (B.5) neuronal activation and (B.6) CD8 T cell development datasets.

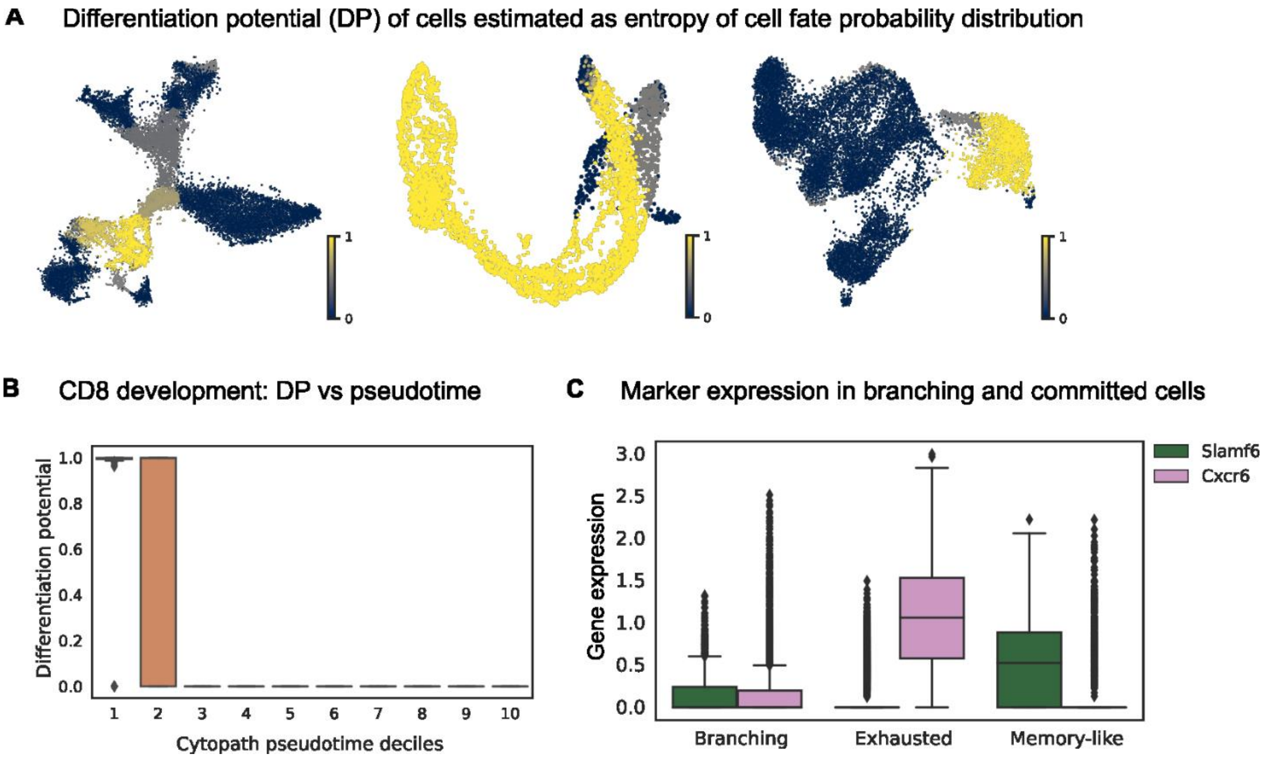


**Figure S6: Branch point detection using cell fate estimated based on relative alignment of cells. Related to STAR Methods.** (A) Differentiation potential per cell of Dendate gyrus, pancreatic endocrinogenesis and CD8 development datasets estimated as the entropy of cell fate probabilities across terminal states and scaled to range [0,1]. (B) Differentiation potential of cells in the CD8 development dataset vs Cytopath pseudotime indicates a branching region (DP > 0) and committed (DP == 0) region. (C) Expression of lineage relevant markers in branching cells (DP > 0) and cells committed to either lineage (DP == 0).

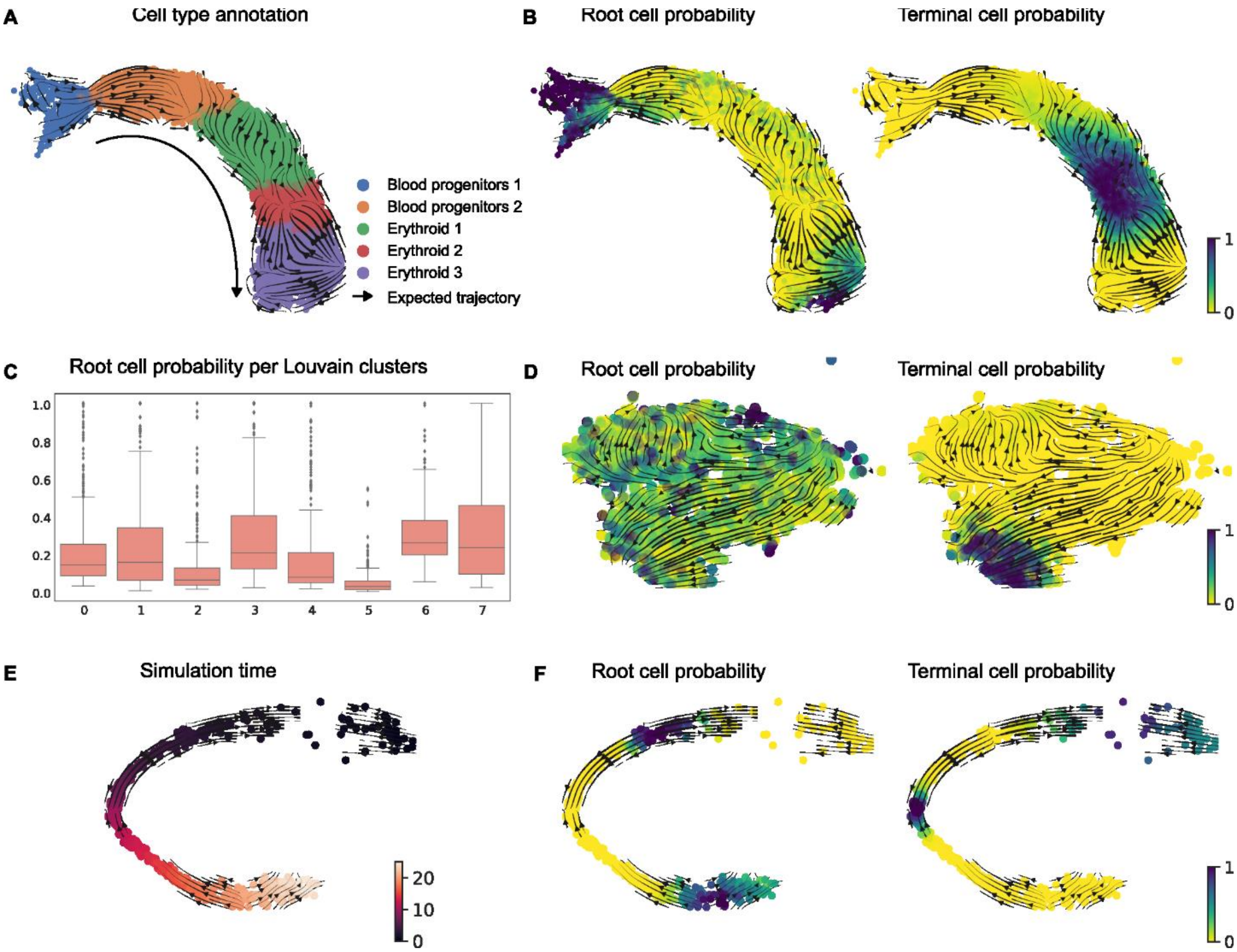


**Figure S7: Root and terminal state detection when RNA velocity fails to capture the true dynamics. Related to STAR Methods.** (A-B) UMAP embedding of the Erthryoid gastrulation dataset overlayed with velocity (stream plot) annotated with (A) cell types and (B) Root and terminal cell probabilities estimated using RNA velocity. (C) Distribution of root cell probability over Louvain clusters for the Hepatocyte zonation dataset. (D) tSNE embedding of the hepatocyte zonation dataset overlayed with velocity (stream plot) and annotated with root and terminal cell probabilities estimated using RNA velocity. (E-F) PCA projection (1 vs 2) of dataset simulated using scvelo including features with time dependent degradation rates overlayed with velocity (stream plot) and annotated with (E) simulation time and (F) root and terminal cell probabilities estimated using RNA velocity.
